# 4CMenB vaccine induces elite cross-protective human antibodies that compete with human factor H for binding to meningococcal fHbp

**DOI:** 10.1371/journal.ppat.1008882

**Published:** 2020-10-02

**Authors:** Daniele Veggi, Federica Bianchi, Laura Santini, Paola Lo Surdo, Chelsy C. Chesterman, Werner Pansegrau, Nicoletta Bechi, Ying Huang, Vega Masignani, Mariagrazia Pizza, Rino Rappuoli, Matthew J. Bottomley, Roberta Cozzi, Domenico Maione

**Affiliations:** 1 GSK, Siena, Italy; 2 University of Florence, Firenze, Italy; 3 GSK, Rockville, MD, United States of America; University of Oxford, UNITED KINGDOM

## Abstract

*Neisseria meningitidis* serogroup B (MenB) is the leading cause of meningococcal meningitis and sepsis in industrialized countries, with the highest incidence in infants and adolescents. Two recombinant protein vaccines that protect against MenB are now available (i.e. 4CMenB and MenB-fHbp). Both vaccines contain the Factor H Binding Protein (fHbp) antigen, which can bind the Human Factor H (fH), the main negative regulator of the alternative complement pathway, thus enabling bacterial survival in the blood. fHbp is present in meningococcal strains as three main variants which are immunologically distinct. Here we sought to obtain detailed information about the epitopes targeted by anti-fHbp antibodies induced by immunization with the 4CMenB multicomponent vaccine. Thirteen anti-fHbp human monoclonal antibodies (mAbs) were identified in a library of over 100 antibody fragments (Fabs) obtained from three healthy adult volunteers immunized with 4CMenB. Herein, the key cross-reactive mAbs were further characterized for antigen binding affinity, complement-mediated serum bactericidal activity (SBA) and the ability to inhibit binding of fH to live bacteria. For the first time, we identified a subset of anti-fHbp mAbs able to elicit human SBA against strains with all three variants and able to compete with human fH for fHbp binding. We present the crystal structure of fHbp v1.1 complexed with human antibody 4B3. The structure, combined with mutagenesis and binding studies, revealed the critical cross-reactive epitope. The structure also provided the molecular basis of competition for fH binding. These data suggest that the fH binding site on fHbp v1.1 can be accessible to the human immune system upon immunization, enabling elicitation of human mAbs broadly protective against MenB. The novel structural, biochemical and functional data are of great significance because the human vaccine-elicited mAbs are the first reported to inhibit the binding of fH to fHbp, and are bactericidal with human complement. Our studies provide molecular insights into the human immune response to the 4CMenB meningococcal vaccine and fuel the rationale for combined structural, immunological and functional studies when seeking deeper understanding of the mechanisms of action of human vaccines.

## Author summary

Serogroup B meningococcus is a human pathogen causing meningitis and sepsis, especially in infants and adolescents. The recent development of two vaccines (4CMenB, MenB-fHbp) provides an opportunity to reduce disease incidence. One vaccine component is the factor H binding protein (fHbp), which exists in three main variants. On the meningococcal surface, fHbp recruits the human complement-downregulating factor H (fH), allowing the bacterium to evade the host immune system. Analyses of antibodies from vaccinated humans can yield insights into vaccine mechanisms of action. Recently, analyses of the response to 4CMenB vaccination identified thirteen new antibodies able to bind all three fHbp variants. We characterized all 13 antibodies. One of them, 4B3, is the first human antibody reported to effectively mediate bactericidal killing of meningococcal strains expressing each of the three fHbp variants and to compete with human fH for binding to fHbp. We present the three-dimensional structure of 4B3 complexed with fHbp, molecular details that explain how 4B3 can cross-react with all different fHbp variants and why it is so potent. This study provides a deeper understanding of 4CMenB vaccine-induced antibodies, and guidance for the rational design of antigens inducing broadly protective immunity.

## Introduction

*Neisseria meningitidis* is a leading cause of meningitis and sepsis worldwide [1]. Before the introduction of antibiotic or vaccine therapies, 70–85% of meningococcal disease cases were fatal [1]. Meningococcal disease is also associated with severe morbidity including limb amputation, hearing and visual impairment, cognitive dysfunction, seizure disorders, and behavioral problems. Because of rapid disease onset and progression, antibiotic therapy remains ineffective in many cases. Indeed, vaccination is widely considered the best approach and MenB vaccines have been intensely researched and developed over the last twenty years.

Like many other pathogenic organisms, *Neisseria meningitidis* can escape the complement response allowing it to proliferate in the human host. Meningococcus recruits the human complement-downregulating protein factor H (fH), enabling meningococcal survival in human serum [2]. Human fH, one of the most abundant complement components in the blood (plasma concentrations 200–800 μg/ml [3]), is a conserved glycoprotein that inhibits the alternative human complement pathway and contributes to the ability of the immune system to discriminate between self and non-self-elements [4,5]. The binding of human fH to meningococcus therefore plays an important role in disease development.

The first broad-spectrum recombinant protein-based vaccine, 4CMenB, against serogroup B meningococcus (MenB), was approved by the EMA in 2013 for prevention of MenB disease in all age groups, and by the US FDA in January 2015 for use in adolescents. One of the four main components of 4CMenB is the factor H binding protein (fHbp) a 28-kDa surface-exposed lipoprotein. fHbp specifically binds fH, allowing the bacterium to cover its surface and thus mimic the host and evade complement activation [2]. fHbp is found in the genome of >95% of all known meningococcal strains and to date over 1100 different amino acid sequences of fHbp have been identified (http://pubmlst.org/neisseria/fHbp)[6]. fHbp sequences can be classified in three main variants which are generally immunologically distinct; within the fHbp variant groups the sequence identity is usually above 87%, whereas between variant groups the sequence identity can be as low as 62% [7,8]. Preclinical and clinical studies have demonstrated that fHbp elicits a robust bactericidal antibody response in mice, rabbits and humans. Presumably due to the high antigenic variability, the majority of vaccine-elicited monoclonal antibodies (mAbs) against fHbp display variant-group specificity [9–16] and only in a few instances anti-fHbp mAbs are able to cross-react with different variants of fHbp [17–20]. Four murine anti-fHbp mAbs (JAR1, JAR3, JAR5, 12C1) inhibit binding of fH to fHbp due to epitope overlap with the fH binding region [11,12,21,22] but, to date no human mAbs able to compete with fH binding have been reported. Since meningococcal fHbp specifically binds human fH (and not to the murine fH)[23], it has been hypothesized that upon human vaccination fHbp present in the vaccine might form a complex with human fH thus concealing the fH:fHbp binding interface, rendering it inaccessible to the immune system [24, 25]. We sought to evaluate the latter hypothesis.

Previously, an interesting subset of cross-reactive 13 anti-fHbp human mAbs all able to bind variants 1, 2 and 3 was identified in a library of over 100 human Fabs [26] (Antigen binding Fragments) obtained from three healthy adult volunteers immunized with 4CMenB. Herein, we have further characterized the key cross-reactive mAbs for their antigen binding affinity, complement-mediated serum bactericidal activity and ability to inhibit binding of fH to live bacteria. For the first time, we identified potent broadly cross-protective anti-fHbp human mAbs able to compete with human fH binding. To elucidate the molecular basis of this competition and the ability to recognize diverse fHbp variants, we determined the co-crystal structure of fHbp variant 1.1 (v1.1) bound to human Fab 4B3. The novel structural, biochemical and functional data are of great significance because these human vaccine-elicited mAbs are the first reported to inhibit the binding of fH to fHbp, and are highly bactericidal with human complement.

## Results

### Selected human mAbs have high affinity for the three fHbp main variants

From the previously reported screening of 110 recombinant human Fab [26], isolated from peripheral blood mononuclear cells (PBMCs) of three healthy adults immunized with 4CMenB vaccine [18], 13 cross-reactive antibodies were identified, tested in protein array epitope mapping suggesting that mAbs have different binding profiles and finally, the crystal structure of one of them (Fab 1E6) in complex with fHbp v3.28 was determined [26]. Here, to extend the functional characterization of this interesting group of cross-reactive antibodies, the heavy and light chain immunoglobulin variable-region genes were produced in mammalian cells as full IgG1 mAbs. Surface plasmon resonance (SPR) was used to measure the binding affinities and kinetics constants of the purified mAbs for three different variants of fHbp. The binding analysis revealed that all mAbs have moderate to high affinity for the three variants of fHbp, with equilibrium dissociation constant (K_D_) values ranging from 10^−9^ M to 10^−11^ M (Table 1). Overall, the kinetics constants indicated that all mAbs bind the fHbp variants with similar association and dissociation rates, forming highly stable complexes (S1A, S1B and S1C Table and S1 and S2 Figs). The SPR data further confirm that immunization with fHbp v1.1, within the 4CMenB formulation, elicits diverse cross-reactive mAbs able to bind tightly to fHbp variants 1, 2 and 3, thus suggesting that the target epitopes are conserved among the 3 variant groups of fHbp.

**Table 1:** Summary of mAbs binding affinity by SPR analysis. Data are shown for all twelve cross-reactive mAbs.

	K_D_ (M)		
mAbs	fHbp v1.1	fHbp v2.16	fHbp v3.28
**3B7**	**2,6E-9 ± 0,06**	**5,51E-10 ± 0,49**	**2,2E-9 ± 0,3**
**1D1**	**8,93E-10 ± 1,03**	**2,61E-9 ± 0,37**	**1,72E-9 ± 0,01**
**3F1**	**1,04E-9 ± 0,05**	**1,13E-8 ± 0,06**	**1,80E-9 ± 0,01**
**1E10**	**1,54 E-9 ± 0,05**	**1,4E-9 ± 0,4**	**1,27E-9 ± 0,02**
**2G1**	**1,08E-9 ± 0,08**	**1,7E-9 ± 0,2**	**1,75E-9 ± 0,01**
**5F12**	**1,01E-9 ± 0,02**	**2,75E-9 ± 0,15**	**1,75E-9 ± 0,01**
**2C8**	**3,4E-10 ± 0,68**	**5,25E-11 ± 0,03**	**4,6E-10 ± 1,6**
**5C6**	**1,08E-9 ± 0,02**	**1,7E-9 ± 0,01**	**2,03E-9 ± 0,02**
**4B10**	**2,96E-10 ± 0.08**	**4,51E-10 ± 0,25**	**1,67E-9 ± 0,03**
**4F9**	**7,7 E-11 ± 1,2**	**2,85E-11 ±0,02**	**2,4E-10 ± 0,9**
**4B3**	**3,28E-10 ± 0,25**	**2,9E-11 ± 0,35**	**6,33E-10 ± 0,13**
**3G7**	**3,44E-10 ± 0,32**	**5,26 E-11 ± 0,22**	**6,8E-10 ± 1,4**

### mAbs recognize native fHbp on live meningococcus

**Fig 1:**
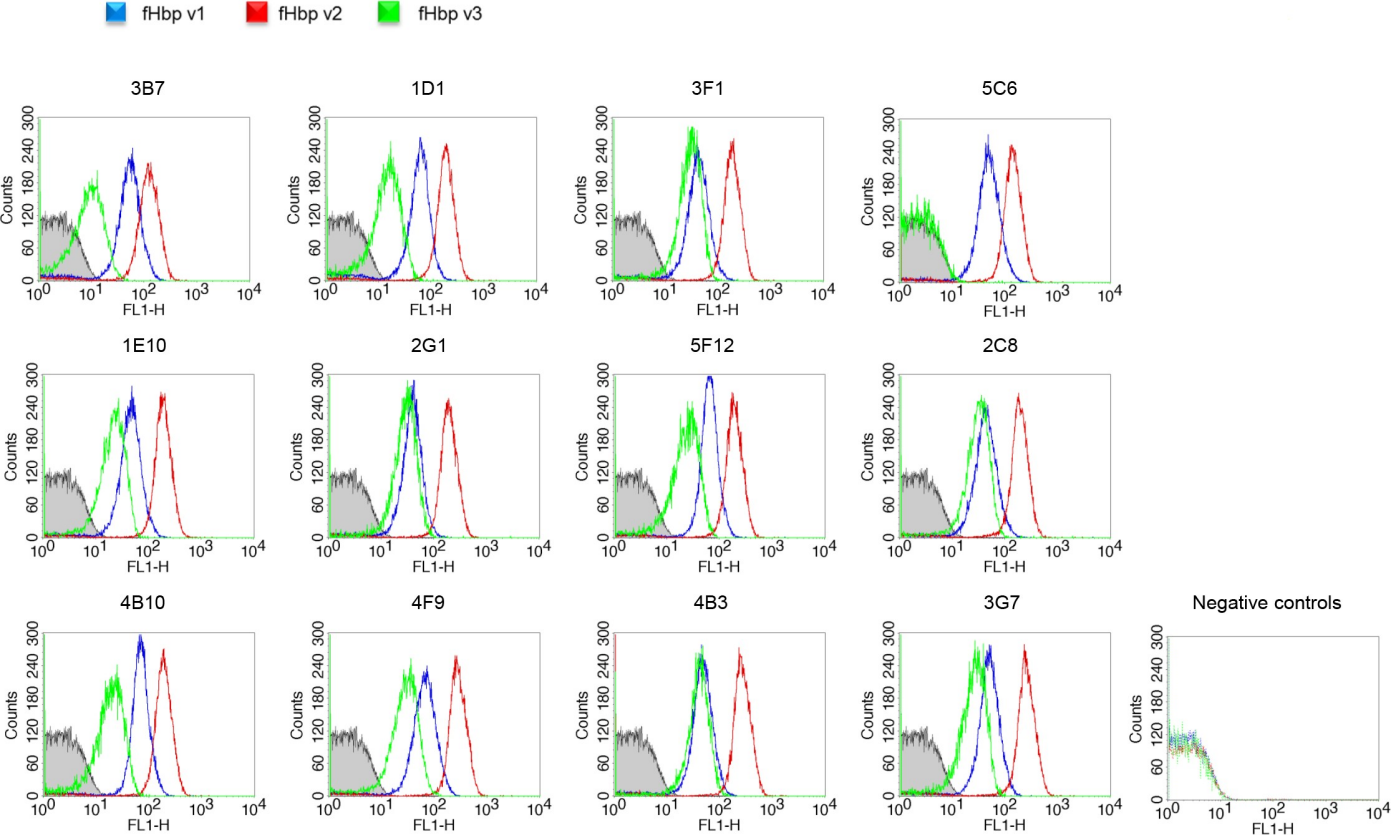
Binding of mAbs to live meningococci as measured by flow cytometry. In blue the MC58 expressing v1, in red the UK104 expressing v2 and in green the UK320 exposing the v3. Gray-filled area represents negative control bacteria incubated with PBS and secondary antibodies FITC-conjugated antibodies.

Since fHbp is a surface-exposed meningococcal lipoprotein, flow cytometry represents a suitable tool to detect the ability of mAbs to bind their full-length target antigens directly on the surface of live bacteria. Therefore, fluorescently-labeled anti-fHbp mAbs were incubated with suspended meningococcal cells, separately carrying three different variants of fHbp, followed by flow cytometry analysis. All 13 human anti-fHbp recombinant mAbs bound native fHbp on the surface of MenB strains MC58, UK104 and UK320, respectively carrying the fHbp v1.1, v2.16 and v3.45 (also indicated herein as v1, v2 and v3) (Fig 1). The only exception was mAb 5C6, which showed no binding to bacteria expressing fHbp v3.45. mAbs showed different extents of binding: 10-fold higher for the meningococcal strain expressing fHbp v2, compared with the strains expressing v1 or v3, likely reflecting the higher amounts of fHbp expressed by the strain carrying v2[27]. These data demonstrate that multiple human mAbs, elicited by vaccination with soluble fHbp v1.1 antigen, are able to recognize diverse fHbp variants 1, 2 and 3 displayed on live serogroup B meningococci.

### Cross-protective bactericidal activity of vaccine-elicited anti-fHbp human mAbs

**Fig 2:**
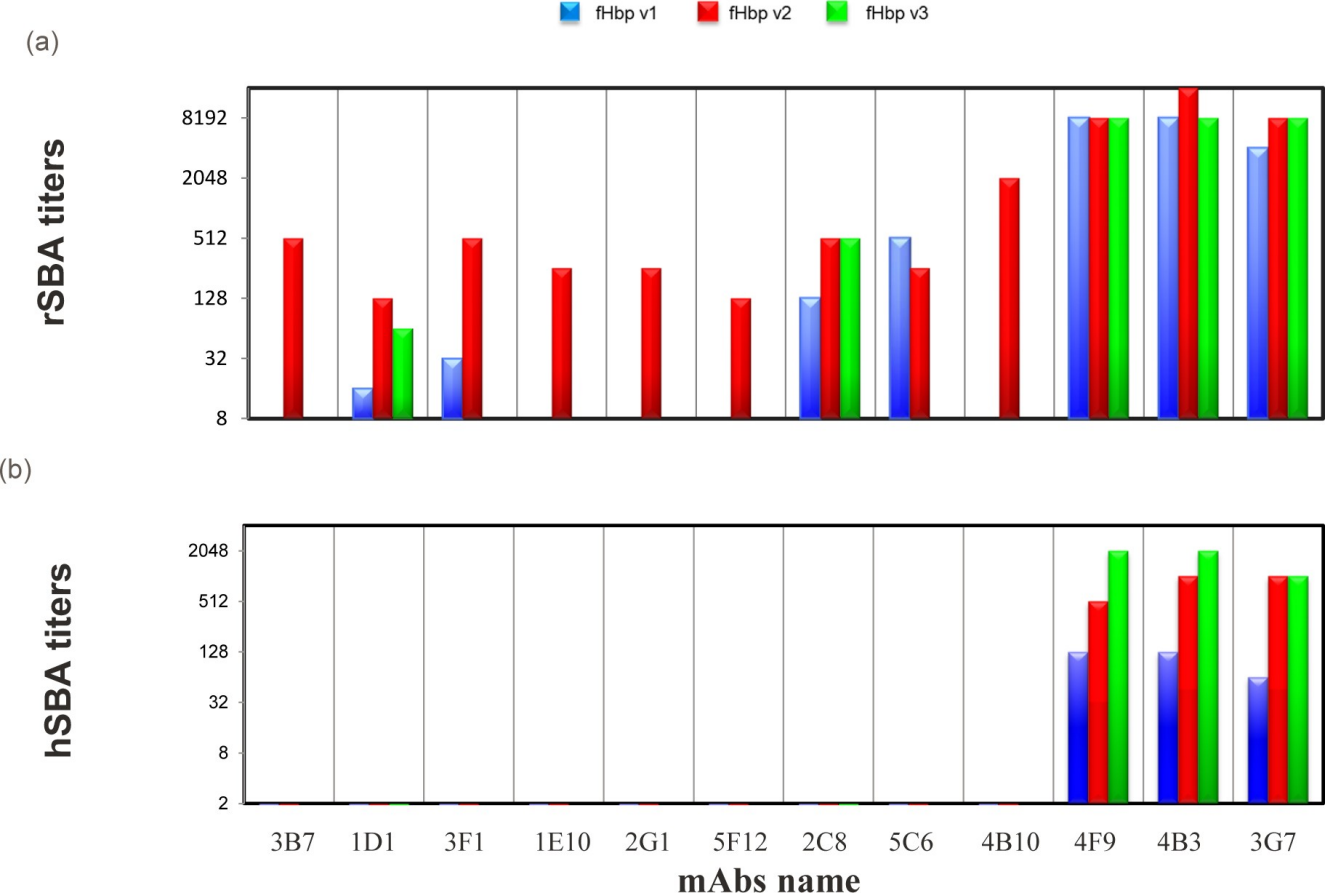
Histogram of bactericidal assay (SBA) titers using rabbit (rSBA) (a) and human (hSBA) (b) serum as source of complement against meningococcal strains expressing v1, v2 and v3 colored in blue, red and green respectively.

The functionality of the set of cross-reactive human mAbs was tested against MenB strains expressing distinct fHbp v1, v2 and v3 with serum bactericidal activity (SBA) assays using baby rabbit serum (rSBA) or human serum (hSBA) as source of exogenous complement. Both serum bactericidal assays are standardized and validated methods to evaluate the immune response to *Neisseria meningitidis* [28] and are widely used as surrogates of protection mediated by antibodies elicited by meningococcal vaccines [29, 30]. The rSBA analysis revealed that seven of twelve mAbs were bactericidal against the strains expressing v1 and five against v3, while all twelve anti-fHbp antibodies were bactericidal against the strain expressing v2. A large difference was observed in the bactericidal titers (which ranged from 16 to >8000) both between different mAbs and between the MenB strains harboring different variants (Fig 2A and S2 Table).

In hSBA assays, three of 12 mAbs tested were able to induce potent killing of all the MenB strains carrying the three different variants of fHbp (Fig 2B). This finding is remarkable, since no single human mAb was previously identified as being able to mediate high titers of bactericidal activity when used alone; rather, only a few exhibited high titers of hSBA when used in combination [11, 12, 18, 21, 22, 31].

### Three mAbs compete with human factor H for binding to fHbp

**Fig 3:**
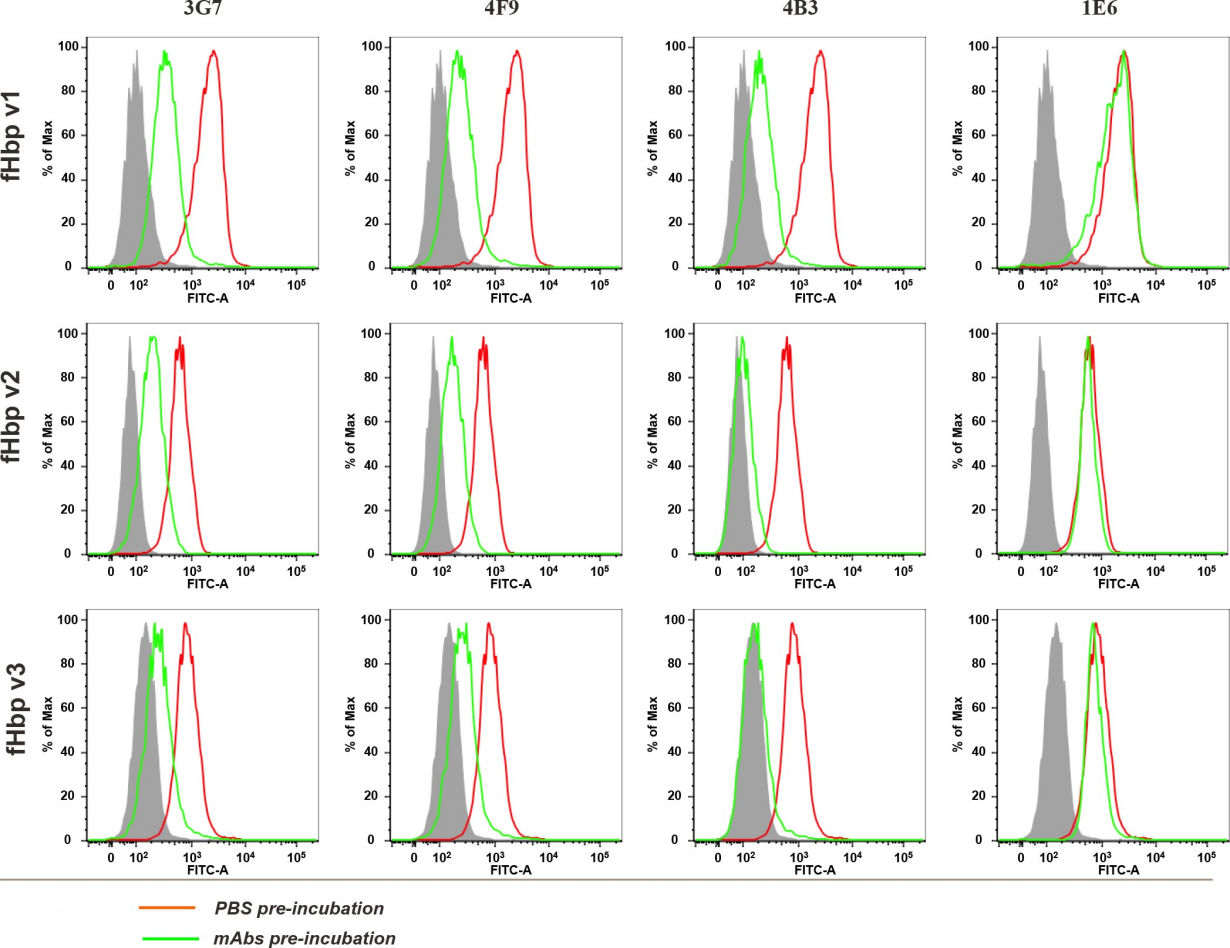
**Flow cytometry binding of fH pre-incubation with PBS (red line) and pre-incubation with mAbs (green line).** Gray-filled area represents negative control bacteria incubated with PBS and secondary antibodies FITC-conjugated antibodies. mAbs 3G7, 4F9 and 4B3 are able to inhibit fH binding, while mAb 1E6 is an example of those not competing with fH binding.

Previous evidence obtained from studies with murine antibodies showed that only those able to inhibit binding of hfH to fHbp had bactericidal activity when tested with human complement [12]. The different degree of SBA functionality elicited by the human mAbs tested in this study suggests that these mAbs might recognize different epitopes on fHbp. To date, no human mAb has been described as able to compete with human fH (hfH) for binding to fHbp, perhaps due to the small number (~10) of human antibodies hitherto analyzed. To explore whether a correlation could be found between SBA functionality and capability to inhibit fH binding, the cross-reactive anti-fHbp mAbs were tested for their ability to inhibit fH binding to native fHbp by flow cytometry analysis. Interestingly, we found that only three mAbs (4F9, 4B3 and 3G7) were able to significantly inhibit hfH binding on meningococcal strains MC58, UK104, M1239, representative of the three variants fHbp v1.1, v2.16 and v3.28, respectively (Fig 3), and that these three mAbs are those found to be cross-bactericidal in hSBA. These remarkable results represent the first evidence that anti-fHbp mAbs able to compete with hfH for binding to fHbp on live serogroup B meningococci can be raised in humans upon immunization with recombinant fHbp v1.1.

### Co-crystal structure defines cross-protective epitope on fHbp v1.1

We sought to obtain molecular details on the mechanism of action of the highly functional mAbs through x-ray crystallography studies. A sequence alignment of 4B3, 3G7 and 4F9 shows that all CDR residues in both heavy and light chains of these potent human mAbs, are highly conserved except for position 91 of VL CDR3, where 4B3 and 3G7 have a tyrosine, replaced by histidine in 4F9 (S3 Fig). Considering the very similar K_D_ values observed for all three mAbs against the fHbp main variants (Table 1) as well as the same functional activity (Figs 2 and 3), it is likely that all CDR residues of 4F9 and 3G7 behave like those defined in the crystal structure of the complex formed by fHbp and Fab 4B3. An analysis of the somatic mutations in the CDRs, performed using the IMGT database [32, 33] revealed that five of sixteen total mutated CDR residues are directly involved in the epitope interactions; i.e. only five residues making interactions with the epitope were not present in the inferred germline precursor (Table 2).

**Table 2:** V-REGION mutations analysis.

***CDR1***					***CDR1***					
**VL_4B3**	S28>M	V29>I	S32>P	Y33>F	**VH_4B3**	T33>P				
**VL_3G7**	S28>M	V29>I	S32>P	Y33>F	**VH_3G7**	T33>P				
**VL_4F9**	S28>M	V29>I	S32>P	Y33>F	**VH_4F9**	T33>P				
										
***CDR2***					***CDR2***					
**VL_4B3**	*NO MUTATIONS*				**VH_4B3**	S52>N	S53>N	S56>N	T57>V	I59>K
**VL_3G7**					**VH_3G7**	S52>N	S53>N	S56>N	T57>V	I59>K
**VL_4F9**					**VH_4F9**	S52>N	S53>N	S56>N	T57>V	I59>K
										
***CDR3***					***CDR3***					
**VL_4B3**	Q91>Y	G93>D	SG94>D	Y97>F	**VH_4B3**	S102>R	Y103>F			
**VL_3G7**	Q91>Y	G93>D	SG94>D	Y97>F	**VH_3G7**	S102>R	Y103>F			
**VL_4F9**	Q91>Y	G93>D	SG94>D	Y97>F	**VH_4F9**	S102>R	Y103>F			

Details of somatic mutations of CDRs. For both Variable Light (VL) and Variable Heavy (VH) chains, were reported the original residue and position, replaced by the somatic mutation (Xnb>Z). The dark-gray highlighted mutations represent the residues directly involved in the epitope interactions. Within the CDRs we observe a marked number of replacements on CDR2 of VH, where polar serine is substituted by longer polar asparagine, hydrophobic isoleucine by potentially charged lysine and polar threonine by hydrophobic valine. Remarkably the Q91 on CDR3 of 4B3 and 3G7 of VL has been replaced by tyrosine, while in 4F9 we find a histidine; a semi-conservative replacement not affecting epitope-paratope relationships.

Based on this information, we selected only mAb 4B3 for large-scale expression as recombinant Fab in mammalian cells, for use in co-crystallization trials with recombinant fHbp v1.1. Crystals were obtained after seven days in buffer containing 0.1M HEPES with 20% w/v jeff ED-2001 precipitant at pH 6.5. The x-ray diffraction data were readily processed and the crystal structure of human Fab 4B3 and fHbp v1.1 complex was determined using the molecular replacement method (Table 3). Electron density maps were of high quality and allowed unambiguous model building and structure refinement to a final resolution of 2.4 Å.

**Table 3:** X-ray data collection and refinement statistics.

	fHbp (v1): 4B3 complex (PDB code 6XZW)
**Crystal**	
Space group	P12_1_1
Cell dimensions	
*a*, *b*, *c* (Å)	47. 34, 90.4, 97.94
α, β, γ (°) **Data collection** Beamline Wavelength (Å)	90, 98.81, 90

	ESRF ID30A-1 0.966
Resolution (Å) Total reflections Unique reflections	46.78–2.39(2.485–2.39) *
	100758 (5753)
	29802
*R*_merge_ *R*_meas,_	0.046 (0.581)
	0.055 (0.684)
*I/σ*(*I*)	16.5 (2.2)
*CC*_1/2_	0.999 (0.822)
Completeness (%)	93.0 (100.0)
Redundancy Wilson B-factor (Å)	3.4 (3.6)
	50.42
**Refinement**	
Resolution (Å)	46.7–2.39
No. reflections	29789
*1R*_work_ / *R*_free_	19.94 /25.43
No. atoms	
Protein	5061
Ligand/ion	12
Water	72
*B* factors	
Protein	55.6
Water	72
R.m.s. deviations	
Bond lengths (Å)	0.01
Bond angles (°) Clash scores Ramachandran Favored (%) Allowed (%)	1.17
	8.0

	94.6
	5.2

* Values in parentheses are for highest-resolution shell.

R_sym_ = Σ hkl Σ i jIi(hkl) − <I(hkl)>j/Σ hkl Σ i Ii(hkl). R_work_ = ΣjjF(obs)j − jF(calc)jj/Σ

jF(obs)j. R_free_ is the same as for R_work_ but calculated for 5% of the total

reflections that were chosen at random and omitted from refinement.

**Fig 4:**
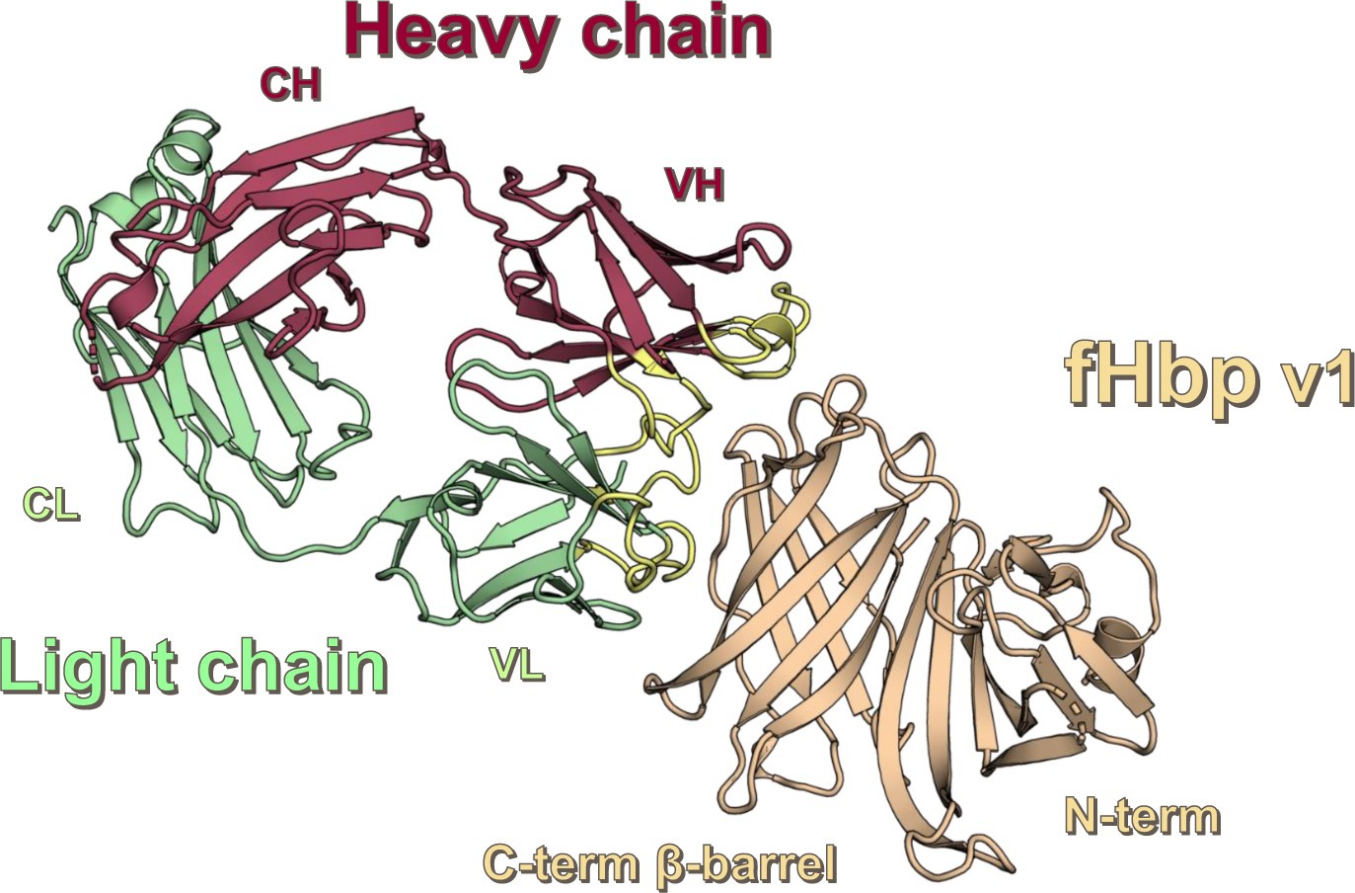
Crystal structure of Fab 4B3 in complex with the fHbp v1 (wheat). The heavy chain and the light chain of the Fab 4B3 are depicted as cartoons, colored in raspberry and pale green respectively. The CDRs of both variable light chain (VL) and variable heavy chain (VH) are colored pale yellow.

**Fig 5:**
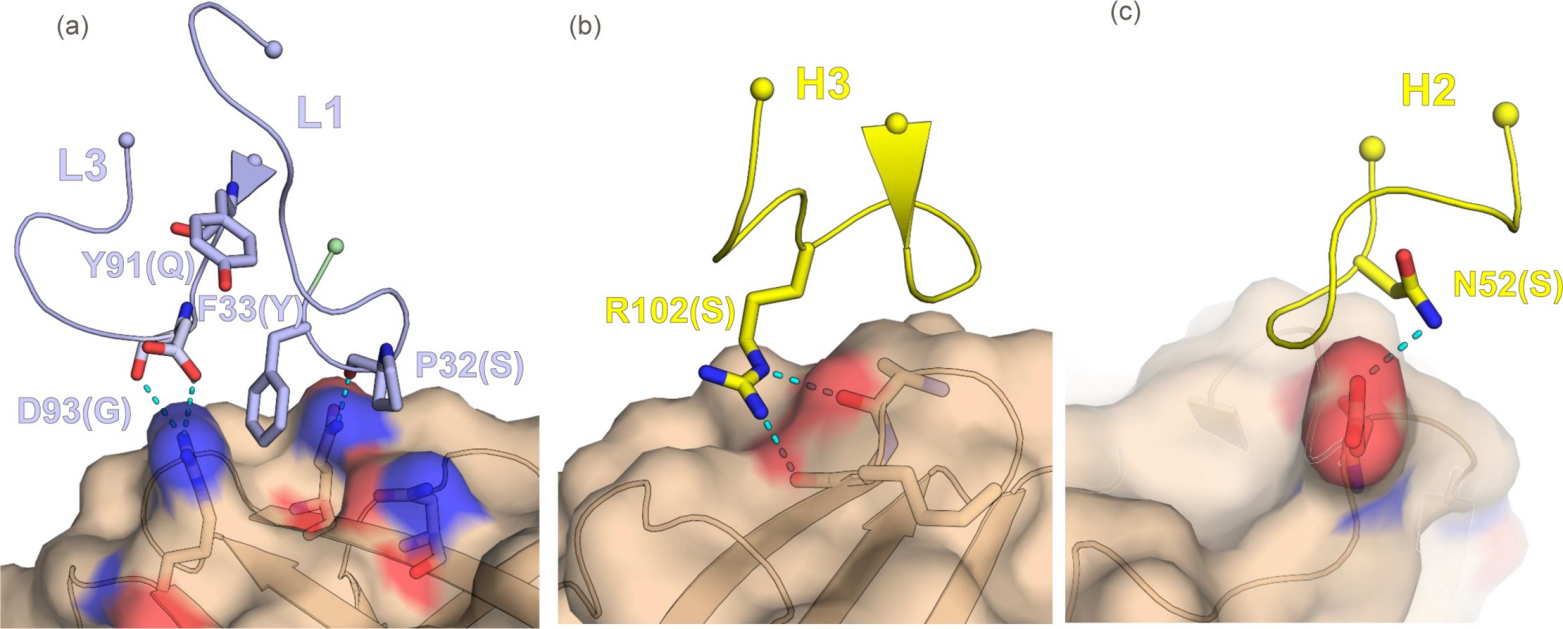
CDRs residues which are directly involved in fHbp interactions. Spheres are the edge of CDRs except for the pale green which represents the first N-term extra-CDR1 residue. In brackets reported the original residues replaced by somatic mutations. Panel (a) shows, P32, F33 and D93 of CDR1 and CDR3 of Variable Light (VL) respectively, engaging constructive interactions. Y91 is the only residue not conserved within all CDRs and has no role in the interaction. In case of Variable Heavy (VH) CDR2 and CDR3 (panel b and c), N52 and R102 contribute respectively to strengthen the network of interactions. these observations suggest an enforcement of the overall interactions with respect to the germline pattern.

The crystal structure of the complex reveals that the Fab 4B3 binds a novel discontinuous epitope located on the C-terminal domain of fHbp (Fig 4), and that the overall structure of fHbp is unaffected by the interaction with the Fab. The Fab 4B3 has the canonical β-sandwich immunoglobulin fold. All six Complementarity Determining Region (CDR) loops are projected towards the C-terminal β-barrel of fHbp, whereas the fHbp N-terminal region is not contacted (Fig 5).

The crystal structure provides a map of the antibody–antigen interface at near-atomic resolution. The structure of the complex reveals that fHbp forms extensive interactions with both variable chains of Fab 4B3 (Table 4). The interaction buries a total area of 847 Å^2^ on the antigen, which is in the typical range for a Fab footprint [34, 35]. The heavy and light chains contribute equally to the buried interface (455 Å^2^ and 391 Å^2^ respectively). The antigen-binding site of the antibody is formed by all six CDRs (Table 3), approximately 15 mAb residues in total are involved in the interaction network.

**Table 4:** Molecular interactions between key residues constituting the Fab 4B3:fHbp v1 epitope.

fHbp v1	huFab 4B3	CDR	Bond type
**F141** **F141**	**S31** **Y32**	**H1** **H1**	**Van der Waals** **Van der Waals**
**D142**	**S31**	**H1**	**hydrogen bond**
**E146** **E146**	**N52** **S54**	**H2** **H2**	**hydrogen bond** **hydrogen bond**
**R149**	**D93**	**L3**	**salt bridge**
**D171**	**F33**	**L1**	**Van der Waals**
**A173** **A173** **A174**	**R102** **N99** **R102**	**H3** **H3** **H3**	**hydrogen bond** **hydrogen bond** **Van der Waals**
**K175** **K175**	**S31** **N99**	**H1** **H3**	**hydrogen bond** **hydrogen bond**
**Q176** **Q176** **Q176** **Q176**	**Y92** **Y92** **P32** **R102**	**L3** **L3** **L1** **H3**	**hydrogen bond,** **Van der Waals** **Van der Waals** **hydrogen bond**
**A195**	**T54**	**L2**	**hydrogen bond**
**D197** **D197**	**R102** **Y50**	**H3** **L2**	**salt bridge** **hydrogen bond**
**K199**	**T57**	**L2**	**hydrogen bond**
**Q216**	**R64**	**-**	**hydrogen bond**
**R204**	**P28**	**H1**	**Van der Waals**
**E218**	**R55**	**L2**	**salt bridge**

### A subset of conserved fHbp residues drives binding affinity in the conformational epitope

**Fig 6:**
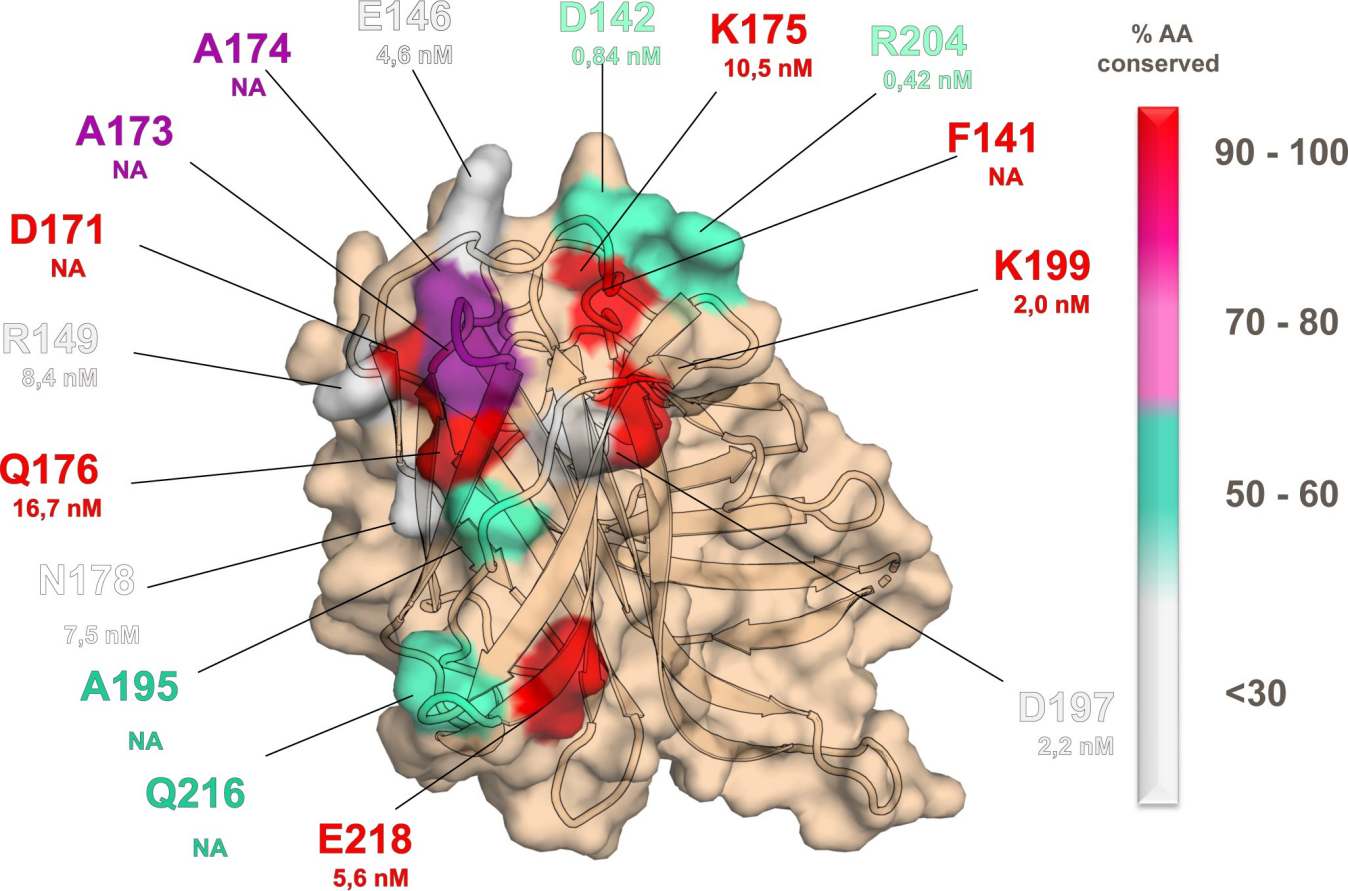
Allelic conservation of the key residues of fHbp v1 engaged in the binding with Fab 4B3. The figure shows the C-terminal β-barrel of fHbp v1 and the surface of the epitope of the Fab 4B3. Each residue is colored based on its conservation among the fHbp gene repertoire which includes 1161 allelic sequences. Where available, the K_D_ value of each single alanine mutant of fHbp has been added.

Fab 4B3 targets a conformational epitope, composed by two distinct regions, the greater of which is mainly defined by heavy chain CDR interactions, while the smaller involves only light chain contacts. By combining visual inspection and computational interface analysis (using PDBePISA)^40^, the fHbp epitope appears to be composed of 15 residues, including hydrophobic, polar and charged amino acids (Fig 6). To better understand the antigen-antibody interface, we performed an extensive alanine scanning study, in which epitope residues were individually mutated to alanine. Mutant fHbp proteins were purified and tested in a biolayer interferometry (BLI) assay for binding to mAb 4B3. Prior to the BLI assay, the thermostability of each purified mutant was measured by differential scanning fluorimetry (DSF). The fHbp wild-type and all mutants tested exhibited melting temperatures (Tm) of at least 75°C (S3 Table), confirming that the mutations introduced had not caused complete unfolding of the proteins. Intriguingly, the alanine mutations had various effects on the strength of interaction, ranging from decreased binding, to no change, to increased binding (Table 5 and S4 Fig).

**Table 5:** Impact of fHbp alanine point mutations on binding to mAb 4B3.

	fHbp Mutation	K_D_ ± SD (nM)	Fold Change
Loss of Function Mutations	Q176A	16.6 ± 2.5	9.8
	K175A	10.5 ± 0.9	6.2
	R149A	8.4 ± 2.1	4.9
	N178A	7.5 ± 0.9	4.4
	E218A	5.6 ± 1.1	3.3
	E146A	4.6 ± 0.5	2.7
Neutral Mutations	D161A	2.5 ± 0.5	1.5
	L213A	2.3 ± 0.2	1.4
	D197A	2.2 ± 0.3	1.3
	K199A	2.0 ± 0.2	1.2
	Wild Type	1.7 ± 0.4	1.0
Gain of Function Mutations	D142A	0.84 ± 0.05	0.5
	R204A	0.42 ± 0.06	0.25

Data are tabulated as the ‘fold change’ in K_D_ value compared to wild type fHbp v1.1, as determined by BLI assay. Note, the D161A mutation was included as a control (residue D161 lies far outside the epitope).

Six mutations that resulted in a notable loss of affinity (K_D_ values increased by 3- to 10-fold) indicated important roles in binding for residues E146, R149, K175, Q176, N178 and E218. The largest differences were conferred by mutations R149A (loss of salt bridge), K175A (loss of H-bond) and Q176A (loss of H-bond), and these residues all appear somewhat clustered in a local surface hotspot (Fig 6). In fHbp variants 1.1, 2.16 and 3.28, residue 149 is always positively charged (either R or K), and both K175 and Q176 are fully conserved, likely promoting the tight binding of 4B3 to all three variants tested. Indeed, K175 and Q176 are conserved at 99.9% in over 1100 different fHbp alleles present in the meningococcal database^6^. In contrast, the R204A mutant bound to 4B3 with 4-fold greater affinity than wild type. In the crystal structure, residue R204 appears only peripherally located at the epitope-paratope interface (closest contact is 4Å to the side chain of Fab heavy chain residue P28). The fHbp R204A mutation eliminates its charged side chain and increases local hydrophobicity, which may facilitate its approach to the uncharged P28. Another group of mutants had a neutral impact on binding affinity, based on a K_D_ change of less than 2-fold compared to the wild type protein. This group included the control mutation D161A, which is intentionally far from the 4B3 epitope, and two mutants on the periphery of the epitope, L213A and K199A. The final member of this group is D197A, where in the wild type protein, D197 was shown to form a salt bridge with R102 in Fab 4B3. While in other locations (E218, R149 for example) a measurable decrease in affinity is seen upon the loss of a single salt bridge, the D197A mutation likely has only a minimal impact because R102 in 4B3 is also positioned to form a salt bridge with 4B3 residue D106. Collectively, this structure-based mutational analysis provides a detailed understanding of the key residues responsible for the high affinity binding of 4B3 to fHbp v1.1.

### Molecular insights into the cross-reactivity of mAb 4B3

As described above, mAb 4B3 binds with high affinity to fHbp main variants 1, 2 and 3 (Table 1). The co-crystal structure presented here reveals the conserved epitope features that underlie the cross-reactivity with mAb 4B3. In particular, the epitope residues F141, D171, Q176, K199, E218 are conserved in >99% of alleles, and indeed residue K175 was present in 100% of alleles (S4 Table and Fig 6). Also, noteworthy, residues A173 and A174 are present in >75% of the sequences. The remaining key residues identified as important for mAb 4B3 binding are localized around the periphery of the epitope and show more substantial sequence variability. Overall, the very high affinity of mAb 4B3 for wild type fHbp v1.1 derives from a combination of interactions mediated by numerous conserved fHbp residues. Indeed, notwithstanding the high antigenic variability of fHbp in general, we show here the structural details revealing how mAb 4B3 is able to efficiently recognize different fHbp variants, suggesting a high potential cross-protection to several meningococcal B strains, conferred by this vaccine-elicited human mAb and the closely related mAbs 4F9 and 3G7.

### Molecular basis of competition by mAb 4B3 and human fH for binding to fHbp

**Fig 7:**
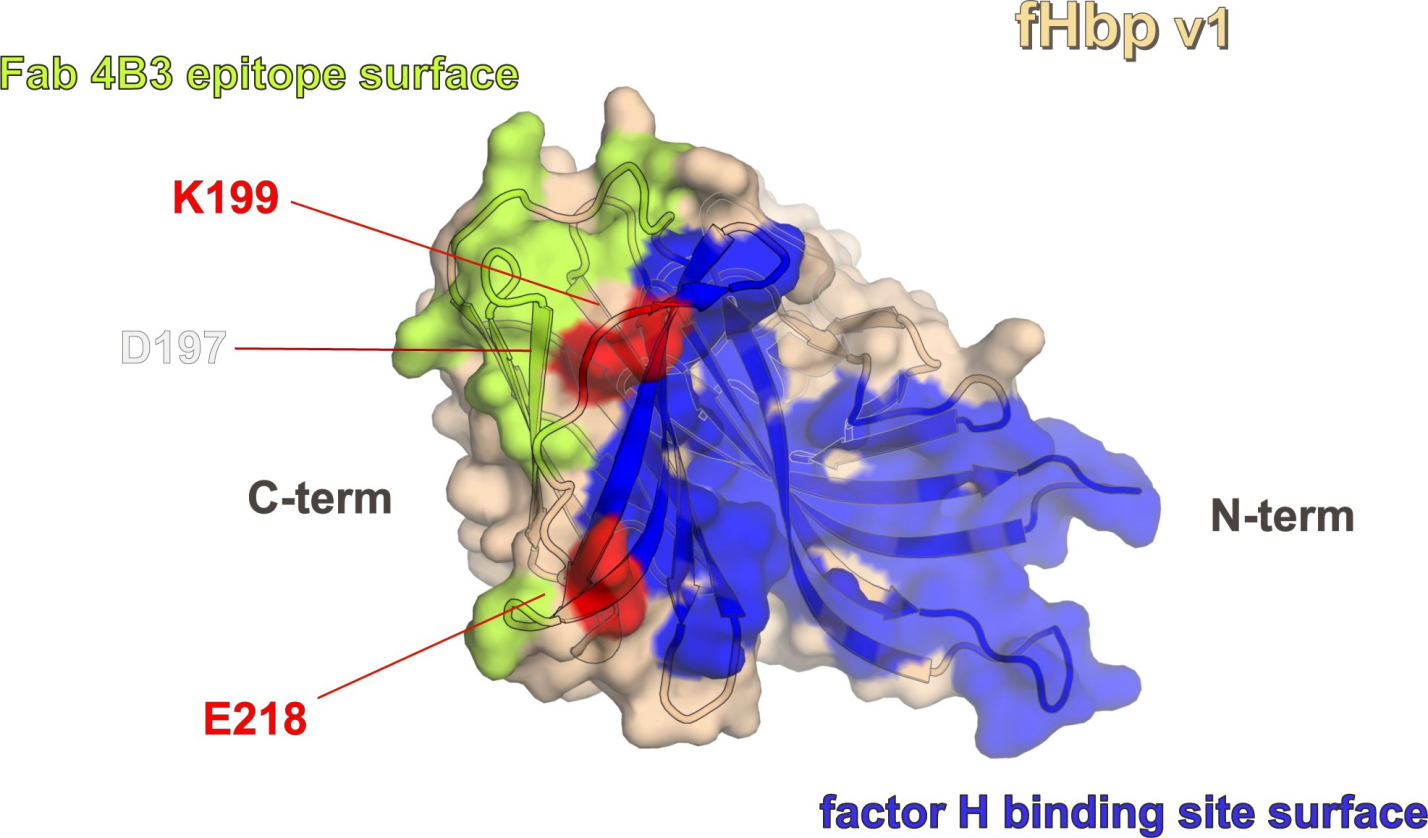
Surface plot of the competition of Fab 4B3 with human factor H for fHbp binding site. The epitope of the Fab 4B3 is represented in lemon instead the human factor H binding site in blue. In red are highlighted the common residues within the two complexes. The label colors indicate the percentage of conservation as reported in Fig 6.

The ability of mAb 4B3 to reduce hfH binding on live bacteria (Fig 3) suggested a competition for the binding site on fHbp. To explore *in silico* this competition, we superimposed the structure of the fHbp/4B3 complex onto known fHbp/hfH complex structures [36,37]. Interestingly, the structural superpositions revealed that the epitope patch on fHbp contacted by the Fab 4B3 light chain overlaps with the region contacted by hfH (Fig 7). A deeper inspection comparing the buried surface areas on fHbp confirmed that the binding sites of the fHbp/4B3 and fHbp/fH complexes overlap at three common residues: D197, K199 and E218, which are involved in both complexes. This spatial overlapping appears to underlie the molecular competition observed, which in turn is likely to explain the remarkably high hSBA titers observed for the three key mAbs studied herein (4B3, 4F9 and 3G7). Residues K199 and E218 are both highly conserved (≥99%) in almost all fHbp sequences known, suggesting that the molecular competition with fH and consequent high bactericidal activity of 4B3 is likely conserved when targeting the vast majority of fHbp variants.

**Fig 8:**
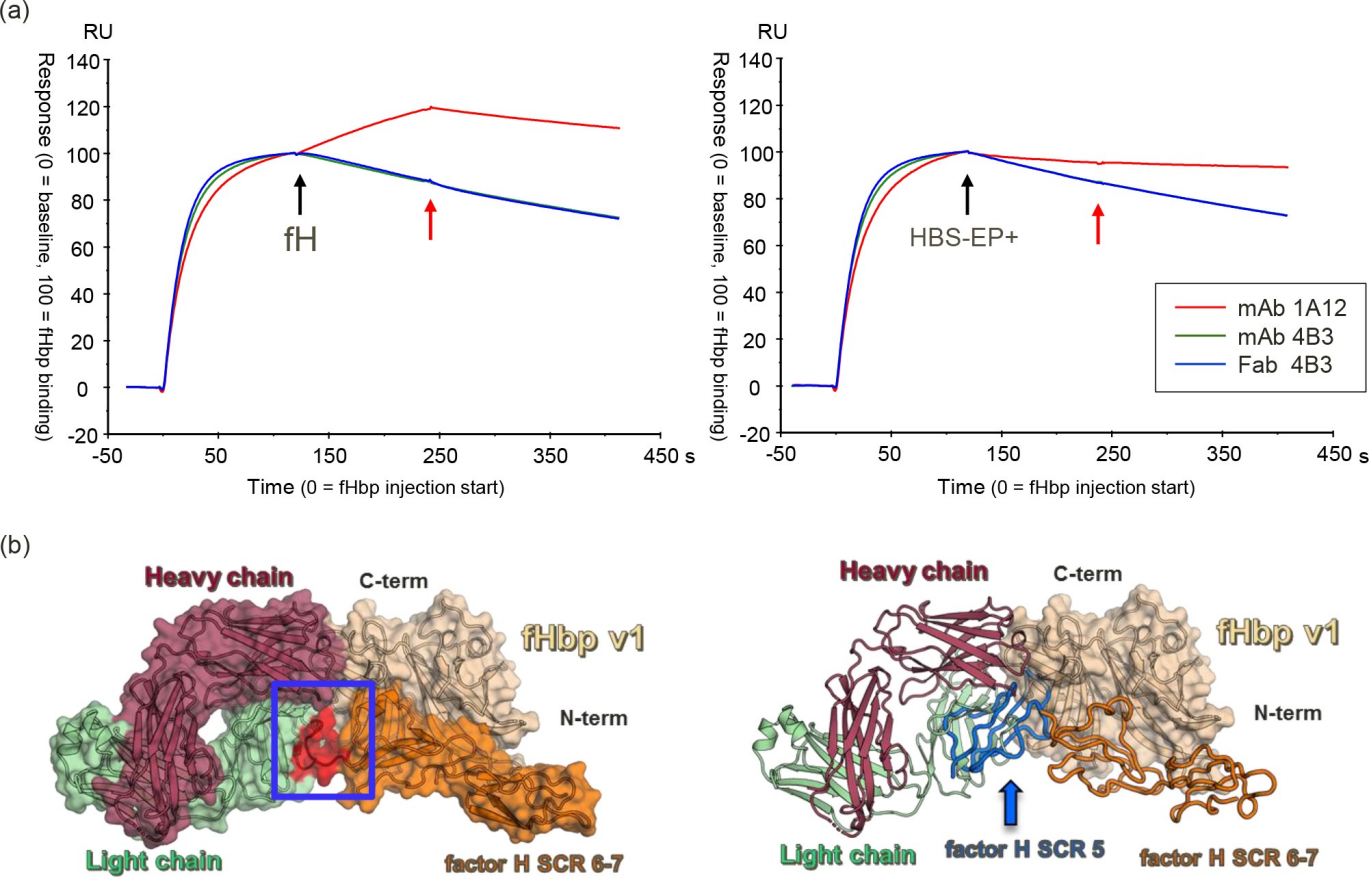
Overall representation of possible accommodations between the complex Fab 4B3/fHbp v1and human factor H. (a) SPR-based factor H competition assay with purified fHbp v1.1 captured on immobilized mAb 4B3, Fab 4B3, and the mAb 1A12. Left panel, start of injection of factor H is indicated by a black arrow, end of injection by a red one. Right panel, buffer control, start of injection of running buffer HBS-EP+ is indicated by a black arrow, end of injection by a red one. (b) On the left superimposition of the crystal structure of Fab 4B3 and the domain 6 and 7 of the human factor H (PDB 2W80) in complex with the fHbp v1. All components are depicted both as cartoons and as solid surface, with the heavy chain (raspberry), the light chain (pale green), the fHbp v1 (wheat) and the human factor H colored in orange. The blue box highlights the region (red) of hfH domain 6 and 4B3 VL overlapping, in the model, suggesting a high likelihood of steric hindrance and clashes within the two proteins in the physiological context. Right, superimposition of the model reported in on the left side and the domain 5, 6 and 7 from X-ray scattering structure of human complement fH (PDB 3GAV) in one of the likely location of domain 5 of fH. In this case we can observe a dramatic collision within domain 5 of fH and human Fab 4B3. The fH domains are depicted as ribbon; while domain 6 and 7 are colored in orange, domain 5 is colored in blue.

To experimentally investigate the nature of the mAb 4B3 inhibition of hfH-binding to fHbp, an SPR competition assay was performed, using recombinant purified fHbp with mAb 4B3, Fab 4B3, and the unrelated mAb 1A12, which does not inhibit binding of hfH to fHbp [19] (Fig 8A) In this experiment, fHbp was captured either on mAb 4B3, 1A12 or Fab 4B3, immobilized on the CM5 SPR sensor surface. By subsequent injection of hfH the ability of captured fHbp to interact with hfH was tested. Binding of hfH to fHbp was totally inhibited in fHbp captured on mAb (or Fab) 4B3. In contrast, capture on mAb 1A12, which binds a distal region of fHbp [19], permitted binding of hfH to fHbp. Since the curves obtained with Fab and mAb 4B3 are very similar, the competition is probably a direct consequence of crucial fHbp residues (D197, K199 and E218) being common to both the hfH binding site and the 4B3 epitope and not due merely to steric hindrance (e.g. by the IgG Fc portion).

## Discussion

4CMenB vaccine is now routinely used in many countries worldwide for prevention of serogroup B meningococcal infection in children and adolescents. Recently, an extensive library encoding over 100 distinct human Fabs against the three recombinant 4CMenB vaccine components was obtained from memory B cells isolated from three immunized healthy volunteers. Here, we sought a more detailed understanding of the human immune response to fHbp present in the 4CMenB vaccine.

The extent of the bactericidal activity depends on the contribution of many factors including the antibody specificity and target binding affinity, the IgG subclass, the kinetics of the reaction, the amount of antigen present on the bacterial surface, as well as the accessibility of the epitope [26]. In addition, anti-fHbp antibodies that blocked binding of fH would be expected to increase susceptibility of the organism to bacteriolysis. The variability of these multiple elements likely underlies the differences in the titers measured between the mAbs and against the meningococcal strains.

Several murine and some human anti-fHbp mAbs have been described in the literature and while three anti-fHbp antibodies elicited in mice were able to inhibit the binding of the hfH to the bacterial surface [11–13, 18, 22, 31], no hfH competitive-antibodies elicited in humans were previously identified. One possible explanation is that the fH binding site is hindered and therefore only partially visible to the immune system upon vaccination, another possibility is that the number of human anti-fHbp mAbs analysed [18] is too low. The analysis of the remaining 100 anti-fHbp human mAbs, which are not cross-reactive among the three main fHbp variants, is still ongoing, and some additional mAbs able to inhibit hfH binding to fHbp v1 have been already identified. Moreover, an analysis of the sequence diversity of fHbp mapped onto the three-dimensional structure (Fig 6) revealed that the hfH binding site corresponds to the most variable region of the protein, making the detection of competitive antibodies harder, which may also explain why the identification of cross-reactive mAbs able to inhibit hfH binding has been unsuccessful so far [17].

In this work, for the first time, we identified three cross-protective human mAbs (4F9, 4B3 and 3G7) able to compete with hfH binding, increasing the susceptibility of the bacteria to the complement mediated bacteriolysis. We found that these three potent mAbs differ by only one amino acid substitution in the VH region and by two amino acids in the VL region.

It has been argued that the fHbp/hfH interaction might skew the human immune response to epitopes distant from the fH binding site [31, 38, 39]. Most of the anti-fHbp MenB-4C-elicited mAbs analyzed in this study appear to not inhibit FH binding. However, in contrast with the hypothesis that upon immunization, the fH binding site on fHbp remains always hindered and therefore not available to the immune system recognition, these new results indicate that at least part of the hfH binding site can be accessible for the elicitation of high-affinity antibodies.

According to previous hypothesis, in the case of sparsely distributed antigens like fHbp, when an anti-fHbp mAb binds to fHbp, the ability of two IgG molecules to engage C1q is limited, thus ultimately resulting in a limited amount of classical complement pathway activation, and low hSBA titers [12]. Moreover, in the case of anti-fHbp mAbs that do not inhibit fH binding, the bound factor H (fH) downregulates complement activation, which limits formation of the membrane attack complex and bacteriolysis. In contrast, if the anti-fHbp mAb can block binding of fH, then the classical pathway activation is sufficient to proceed to bacteriolysis. In the previous study by Giuliani M. et al, on a panel of 12 anti-fHbp mAbs, none of them was able to inhibit the hfH binding and only two mAbs (1G3 and 1A12) showed very low bactericidal activity when tested in hSBA [18]. Here, in line with the proposed hypothesis and with previous observations obtained from studies with chimeric murine mAbs [12, 13], we showed that the only human mAbs with high hSBA titers were those able to compete for fH binding to fHbp.

In order to more deeply characterize these three elite human mAbs and to gain insights into the molecular mechanisms underlying their functionality, the crystal structure of fHbp v1, the variant contained in the 4CMenB vaccine, in complex with the cross-protective anti-fHbp Fab 4B3, able to compete with hfH, was obtained and revealed insights on the molecular mechanisms which confer its high potency. The crystal structure of fHbp v1 in complex with the complement control protein (CCP) domain 6 and 7 of the hfH was previously determined, revealing crucial residues located either in the C-terminal beta-barrel or in the N-terminal domain of fHbp [36]. Superimposing the crystal structures of the complexes of fHbp v1:Fab4B3 and fHbp v1: hfH we observed that they partially overlapped in correspondence of three well conserved residues (K199, E218, and D197) partially sharing the interaction interface (Fig 8B). Moreover, superimposing the crystal structure of the complex with domain 5,6 and 7 of X-ray scattering structure of human complement fH (PDB 3GAV), in one of the likely location, we observe a dramatic collision within the Fab and the domain 5 of fH. Thus, we can conclude that domain 5 presumably has the potential to add to steric occlusion in the full length fH. The SPR experiments using purified components, combined with the structural analyses above, demonstrated that 4B3 competes directly with fH for binding to fHbp under *in vitro* label-free conditions. This direct competition for the three key residues in the epitope is likely further enhanced by significant steric hindrance effects readily appreciated when considering that full-length hfH contains many domains (not just domains 6,7 present in the crystal structure).

To further investigate the structural bases underlying the fHbp-dependent cross-variants antibody response in humans, we used the high resolution information of the complex to define a unique set of residues forming a conformational epitope that was not previously seen in other crystal structures of fHbp complexed with mAbs raised in mice, or humans [19], and which occurs in a completely different region on the C-terminal domain of fHbp v1. Overall, numerous H-bonds, salt bridges and Van der Waals interactions are widely distributed across the binding interface and contribute collectively to the very strong binding observed. In total fifteen antigen residues are involved in the binding and the sequence alignments indicate that most of the key residues are well conserved between the fHbp variants tested. Biochemical assays showed the ability of mAb 4B3 to bind three representative variants of fHbp therefore we assume that residues which contribute to variants recognition are strongly conserved. We extended this analysis on the vast number of fHbp sequences identified from clinical isolates and carrier strains (n = 1119) (BIGSDB)[6], by calculating the degree of residue conservation in the 4B3 epitope. We found that many residues are conserved in more than 99% of all fHbp sequences, suggesting that the antibody 4B3 could have a broad coverage on meningococcal circulating strains. Although additional investigations would be needed to demonstrate the full cross-reactivity of mAb 4B3 toward the many known subvariants, our structural data suggest a wide recognition of the great majority of fHbp antigens, with potential to induce bacterial killing of many diverse meningococcal strains either alone or cooperatively with other mAbs against fHbp or in synergy with antibodies against alternative MenB surface antigens.

Interestingly, the epitope and paratope contact areas assume a reciprocal complementary shape requiring mild conformational adjustments in fHbp which might contribute to reinforce the strength of the binding. Although the fHbp C-terminal β-barrel is usually a well folded, stable and rigid part of the protein, the loops which connected the antiparallel strands confer a certain degree of flexibility to the structure and long-range forces, like ionic interactions, or local hydrogen bonds could lead to the observed conformational changes in fHbp [40].

Overall our findings, although not directly predicting vaccine coverage, reinforce the evidence that vaccination with 4CMenB induces the production of potent anti-fHbp cross-protective antibodies. These antibodies could work in synergy with antibodies directed against other vaccine components to reach the threshold required to properly activate the complement cascade and elicit killing of a wide panel of bacterial strains. Here we describe 4B3, a broadly active human monoclonal antibody which alone kills bacteria harboring either of the three antigenic variants of fHbp, combining two independent functions such as binding to fHbp and preventing factor H binding. Understanding the mechanistic nature of complement mediated bacterial killing is of vital importance for the structure-based design of potent and broadly active vaccines.

## Materials and methods

### Ethics statement

Human samples were collected from three adults immunized with Bexsero, also referred as 4CMenB, in a clinical trial (MENB NEXT GEN-001 (V114_01) 205285/ETR) conducted in Krakow, Poland, approved by the Bioethics Committee of the District Medical Doctors Chamber in Krakow and conducted in accordance with the Declaration of Helsinki. The use of samples was performed upon written informed consent obtained from participants before the study-specific procedures.

### Molecular cloning and protein production of fHbp variants

Full-length ectodomain proteins of v1.1 fHbp (UniProt Q6QCC2) from *N*. *meningitidis* strain MC58, v2.16 (Q6VS19) from strain 961–5945, or v3.28 (Q19KF7) from strain M1239 were prepared using the pET-21b plasmid (Novagen), as described previously [8,32]. Site-directed point mutations in the v1.1 construct were introduced using standard PCR methods with specific oligonucleotides and the KLD enzyme mix method (New England Biolabs). All clones were verified by DNA sequencing.

Unless otherwise stated, fHbp was expressed as recombinant protein with a 6-His tag at the C-terminus in *E*. *coli* strain BL21(DE3)[27]. Cultures were grown in ENPRESSO medium following the manufacturer protocol and protein expression was induced by 1mM IPTG for 24h at 25°C.

*E*. *coli* cells were lysed by cell lytic express (Sigma-Aldrich). The bacterial lysate was centrifuged at 9000 rpm for 30 min. The soluble fraction was filtered by 0.22-micrometer filter (Millipore) to remove cell debris and protein was purified by Ni^2+^-affinity chromatography (Ni-NTA agarose resin, Qiagen), buffer-exchanged to Tris-HCl 10mM, NaCl 300mM, sodium azide (w/v) 0.03%, pH 8 and stored at 4°C. Protein concentration was determined using a nanodrop spectrophotometer (Thermo Scientific) and purity was assessed by SDS-PAGE on a 4–12% Bis-Tris Gel after Problue Safe Stain (Giotto Biotech). When necessary, a second purification step of size exclusion chromatography was performed to remove all the impurities using a HiLoad 26/60 column Superdex 75 prep grade (GE).

Protein samples for biolayer interferometry (BLI) binding assays were produced in medium throughput format using *E*. *coli* strain BL21 Star (DE3) cells (Thermo Fisher) grown in 4 ml TB (terrific broth) media in duplicate using deep 24-well plates. Cell pellets were lysed using BugBuster extraction reagent (Millipore), 1 ml per well of deep 24-well plate. The bacterial lysate was centrifuged at 4,000 rpm for 30 min to collect soluble fraction. For each protein to be purified, 2 ml lysate from duplicate wells of deep 24-well plate was moved into a deep 96-well plate. 6-His tagged fHbp protein was purified in one step by Ni^2+^-affinity chromatography, using MagneHis protein purification system (Promega). Proteins were subjected to SDS-PAGE analysis and purity was assessed using Coomassie blue staining.

### Recombinant mAbs production in mammalian cells

The DNA strings encoding the variable region (V) of the heavy (H) and light (L) chains of human monoclonal antibodies, codon optimized for mammalian expression, were synthesized by Geneart (Life Technologies) adding at the 5’ and 3’ extremities an Eco31I site. Synthetic DNA strings were ligated into an expression vector containing a human Ig gene signal peptide sequence and the Eco31I cloning site upstream of the human IgG1, Igκ or Igλ constant regions developed in-house by standard digestion-ligation protocol using competent cells *E*. *coli* MachI (Lifetech).

The recombinant mAbs were expressed in mammalian cells Expi293 (Thermo Fisher) by transient transfection in suspension, powered by the cationic lipid-based ExpiFectamine 293 transfection reagent in combination with specialized transfection enhancers. To transfect equal amounts (15 μg each/30 ml of transfection volume) of IgH and corresponding IgL chain expression vector DNA were used. Cells were centrifuged at 350 x g for 10 minutes 3 and 6 days after transfection and the supernatant collected and filtered over a 0.22 micrometer filter (Millipore) to remove cell debris.

The recombinant full IgGs were purified by affinity chromatography using protein G Sepharose 4 fast flow (GE Healthcare). According to the manufacturer’s protocol mAbs were eluted with 0.1 M glycine (pH 3.0) in tubes containing 1 M Tris (pH 9.0) to avoid pH shock which could damage the proteins. After elution the antibodies were immediately exchanged into PBS buffer using PD-10 desalting columns (GE Healthcare) and were quantified by absorbance at 280 nm using a NanoDrop spectophotometer (ThermoFisher). Protein purity was assessed by SDS-PAGE after Coomassie staining (Problue Safe Stain, GiottoBiotech) in reducing and non-reducing conditions.

### Recombinant Fabs production in mammalian cells

For the expression of recombinant Fabs the variable regions of the heavy and light chains strings were cloned into an expression vector encoding the Fab constant region fused with a Strep-tag at the C-terminal and expressed in mammalian cells Expi293 (Thermo Fisher) following the procedure described above for mAbs production. To further purify the Fabs, the supernatant was exchanged in PBS buffer by dialysis, loaded onto a StrepTrap HP column and eluted by 2.5 mM desthiobiotin buffer (IBA solution for life). The STREP tag was removed by TEV protease cleavage, at a ratio of 1:100.

### Bacterial strains and culture conditions

*N*. *meningitidis* strains MC58 and M11295 were used as reference strains for fHbp v1, M08-240104 (UK104) was used as reference strain for fHbp v2, M01-0240320 (UK320) and M1239 were used as reference strains for fHbp v3. Bacteria were grown on chocolate agar (Biomerieux 43101) at 37°C, 5% CO_2_ overnight. For liquid cultures, colonies from overnight growth were used to inoculate 7 ml cultures (in MH broth supplemented with 0.25% glucose) to an optical density at 600 nm (OD_600_) of 0.05. The culture was incubated for approximately 1.5 to 2.5 h at 37°C with shaking until early log (OD_600_ of 0.25) or mid-log phase (OD_600_ of 0.5).

### Fluorescence-activated cell sorter (FACS) analysis

The ability of mAb to bind antigen exposed on the surface of *N*. *meningitidis* bacteria was determined using a FACScan flow cytometer. Bacteria grown until mid-log phase (OD_600_ of ~0.5) were incubated with mAbs at the concentration of 10μg/ml. Antibody binding was detected using an Anti-Human IgG (H+L)- fluoresceinisothiocyanate FITC conjugated produced in goat (Jackson Immuno Research 109.096.088) at a 1:100 dilution. Bacteria plus PBS-1%BSA and secondary antibody were used as negative control.

### Serum bactericidal activity assay

Serum bactericidal activity against *N*. *meningitidis* strains was evaluated as reported elsewhere [41]. Bacteria grown until early log phase (OD_600_ of ~0.25) were diluted in Dulbecco Phosphate Buffered Saline (DPBS- SIGMA D8662) containing 1% bovine serum albumin (BSA) and 0.1% glucose at the working dilution of 10^4^−10^5^ CFU/ml and incubated with serial two fold dilutions of test mAb starting from a concentration of 125μg/ml. Serum bactericidal titers were defined as the mAb dilution resulting in 50% decrease in CFU per milliliter after a 60-min incubation of bacteria with the reaction mixture compared to the control CFU per milliliter at time zero. Pooled baby rabbit sera from Cedarlane or human serum, obtained from volunteer donors under informed consent, were used as a complement source for rSBA or hSBA respectively.

### Inhibition of binding of human factor H

The ability of the mi sembra che nel resto del paper vengano sempre indicati come mAb to inhibit binding of fH to live bacteria was measured by flow cytometry. Bacterial cells grown until mid-log phase (OD_600_ of ~0.5) were incubated with anti-fHbp mAb (50 μg/ml in PBS-1%BSA buffer) for 30 min at room temperature, followed by the addition of purified human fH (50 μg/ml for MC58 and M1239, 10 μg/ml for UK104), which was incubated for an additional 30 min at room temperature in a final reaction volume of 100 μl. fH binding was detected with a goat polyclonal antiserum to fH (Calbiochem 341276) diluted 1:100 and incubated for 30 min at room temperature, followed by additional 30 min incubation with a donkey anti-goat IgG–fluoresceinisothiocyanate (FITC) conjugate (Jackson Immunoresearch 705.095.003) diluted 1:100 in PBS-1%BSA buffer.

### Surface plasmon resonance (SPR) binding analyses

A Biacore T200 instrument (GE Healthcare) was used to investigate the interaction between mAbs and the purified recombinant fHbp variants, in terms of binding affinity and association/dissociation kinetics. Immobilization and binding experiment were performed in running buffer HBS-EP+ (10mM Hepes, 150mM NaCl, 3mM EDTA supplemented with 0,05% (vol/vol) P20 surfactant, pH 7.4) at 25°C. A commercially available Human Fab Antibody Capture Kit (GE Healthcare) was used to immobilize an anti-human Fab antibody by amine coupling on a carboxymethylated dextran sensor chip (CM-5; GE Healthcare). A density level yielding ∼9,000 response units (RUs) was prepared for the immobilization on two flow cells of the CM5 chip. To determine binding affinity and kinetic parameters, 800–1200 RU of mAbs were captured on one the flow cells of the sensor chip and five incremental concentrations of fHbp were applied as analyte at a flow rate of 40 ul/min. The concentrations of injected fHbp proteins were adapted for each mAb, ranging from 0.39–6.25 nM to 3.1–50 nM, to achieve a maximum of 200 RU of fHbp. The chip surface was regenerated using 10 mM glycine pH 2.1 (180 seconds, flow rate 10 μl/min). A blank injection of buffer only was subtracted from each curve, and reference sensograms were subtracted from experimental sensograms to yield curves representing specific binding. The data shown are representative of two independent experiments. SPR data were analyzed using the Biacore T200 Evaluation software (GE Healthcare). Each sensogram was fitted with the 1:1 Langmuir binding model, including a term to account for potential mass transfer, to obtain the individual k_on_ and k_off_ kinetic constants; the individual values were then combined to derive the single averaged K_D_ values reported.

To assess competition between mAb and hfH binding to fHbp, mAbs 1A12, 4B3 and Fab 4B3 (20 μg/ml in 10 mM sodium acetate, pH4.5) were immobilized by amine coupling to flow cells (FC) 2, 3, and 4, respectively, of a CM5 sensor chip (GE Healthcare). FC 1 was used as reference after blank immobilization with ethanolamine. fHbp v1.1 (200 nM in HBS-EP+) was applied for 120 s at a flow rate of 30 μl/min to all four FCs, immediately followed by 120 s of 200 nM hfH (Calbiochem) using the dual injection type of program. Dissociation was registered for 180 s after which the chip was regenerated by injection of 10 mM glycine-HCl pH 2.0 for 45 s at 30 μl/min. Reference sensograms (FC 1) were subtracted from active sensorgrams (FC 2–4) to yield curves representing the specific binding.

### Differential scanning fluorimetry (DSF) analyses

Purified fHbp protein samples were diluted in 20 mM HEPES pH 7.0, 150 mM NaCl to an approximate concentration of 400 μg/ml and a total volume of 20 μl. Samples were further diluted with 400 μl of buffer followed by concentration to 50 μl using 0.5 ml, 10 kDa cutoff devices from Millipore (Cat #UFC5010BX). This process was repeated twice to reduce imidazole and other buffer components remaining from the purification. Samples were loaded into high-sensitivity capillaries (Part #PR-C006) and analyzed using a Prometheus NT.48 nanoDSF system (nanoTemper). The melting scan was performed from 24°C to 110°C with a ramp rate of 1°C / min. Duplicate measurements were performed for each sample and melting temperature was determined using the manufacturer provided software.

### Biolayer interferometry (BLI) binding analyses

Mutant protein samples were produced in small scale cultures of 4 ml *E*. *coli* BL21(Star) grown at 30°C for 16 hours. Cells were lysed with BugBuster buffer and proteins were purified in one-step using nickel-affinity chromatography. Protein was quantitated using Biolayer interferometry (BLI) analysis using an Octet 384 Red Instrument (Pall FortéBio) and Anti-HIS (HIS-2) biosensors for quantitation. Highly pure fHbp v1.1 was used for the generation of a standard curve. Biolayer interferometry (BLI) analyses of binding interactions were also performed using the Octet 384 Red Instrument (Pall FortéBio), operating at 30°C. Wild type and mutant fHbp proteins were diluted in Octet buffer (1 x PBS, 1% (w/v) BSA) with protein concentrations ranging from 150nM to 1nM. Binding kinetics were measured using Anti-Human Fc (FortéBio) biosensors to capture the mAb 4B3, and fHbp in solution. For each protein, binding traces (average n = 4) were fit using the FortéBio Analysis 11.0 software to determine K_D_, k_on_ and k_off_. Binding experiments were performed in triplicate and the average value is reported.

### Protein crystallization and diffraction data collection and processing

After overnight incubation at 4°C, the Fab:fHbp complex was purified by size exclusion chromatography using a pre-packed HiLoad 26/60 column Superdex 75 prep grade (GE Healthcare) following manufacturer protocol. After removal of monomeric proteins, the Fab:fHbp dimeric complex was concentrated to 25 mg/ml in 50mM Tris-HCl and was screened in over 800 different crystallization screening experiments. Using a Crystal Gryphon robot (Art Robbins Instruments), each experiment was prepared using 200nl reservoir and 200nl protein sample. The best crystal was grown in buffer containing 0.1M HEPES with 20% w/v jeff ED-2001 as precipitant at pH 6.5. Crystals were soaked in the original mother liquor supplemented with 15% ethylene glycol prior to cryo-cooling in liquid nitrogen.

### Structure determination and refinement

Diffraction of the crystals was performed at beamline ID30A-1 of the European Synchrotron Radiation Facility (ESRF) and several full datasets were collected at 100K, at wavelength λ = 0.983 Å, on a Pilatus 6M detector. Diffraction datasets were indexed and integrated using iMOSFLM and reduced using Aimless, via the CCP4 suite [42]. Crystals of fHbp:Fab4B3 complex belonged to space group P1 21 1.

The structure of the complex was solved at 2.4 Å resolution by molecular replacement with Phaser [43] using as model templates for fHbp the protein data bank (PDB) code 2YPV and for the variable region of the light and heavy chain of Fab 4B3 the PDB code 5I17. Initial molecular replacement solutions were subjected to subsequent cycles of manual building in Coot with Phenix.refine [43]. The buried surface areas and atomic interactions/contacts, and the root mean square displacements, were calculated with PISA [44] and Superpose respectively [45].

## Supporting information

S1 Fig mAbs binding affinity data by SPR analysis (reported in S1 Table) represented and summarized for all three fHbp variants as bar chart. Data are shown for all twelve cross-reactive mAbs.
(DOCX)

S2 Fig SPR sensograms for two mAbs (5C6 and 4B10) for three fHbp variants. (DOCX)

S3 Fig Aminoacidic sequence alignment of VH and VL chains for anti-fHbp cross-reactive mAbs. mAbs 4F9 and 4B3 and mAbs 1E10 and 2G1 have the same VH chain, while mAbs 1D1 and 3F1 have the same VL chain. (a) The nucleotide sequence alignment of VL chains, revealed that they differ by only for five substitutions overall, of which only the positions 9 and 271 introduce different residues within the mAbs. Remarkably, the substitution of thymine 271 in cytosine occurs in the CDR3 replacing the tyrosine on 4B3 and 3G7 with histidine on 4F9. (b) VH chains show five nucleotide substitutions. Only mutation in position 13 introduces a valine residue in 4B3 and 3G7 mAbs, while in 4F9 there is a leucine. The sequence alignment was performed with ClustalW and further represented using ESPript [46].
(DOCX)

S4 Fig Experimental and fitted curves for all mutants tested in BLI experiments. (DOCX)

S1 Table Summary of SPR analysis. Data are shown for all twelve cross-reactive mAbs.
(DOCX)

S2 Table A subset of mAbs was tested in duplicate using rabbit (rSBA) and human (hSBA) serum as source of complement against meningococcal strains expressing v1, v2 and v3. The results in bold, in the first column of each strain, were used for the histograms in Fig 2.
(DOCX)

S3 Table Melting temperature (Tm) of fHbp proteins determined by DSF. Each Tm value provided is the average of two independent experiments.
(DOCX)

S4 Table Degree of conservation of the key residues involved in fHbp v1 and mAb 4B3 binding. The table shows the degree of conservation of the key residues of the mAb 4B3 epitope between fHbp sequences repertoire accessible in the *Neisseria Meningitidis* multilocus sequence typing (MLST) database at the website to https://pubmlst.org/neisseria which includes 1119 alleles of fHbp.
(DOCX)
