# Supplementary figures and images for: 4CMenB vaccine induces elite cross-protective human antibodies that compete with human factor H for binding to meningococcal fHbp
Source: PLoS Pathog. 2020 Oct 2;16(10):e1008882. doi: 10.1371/journal.ppat.1008882 (PMC7556464; doi:10.1371/journal.ppat.1008882)

**S1 Figure**

**
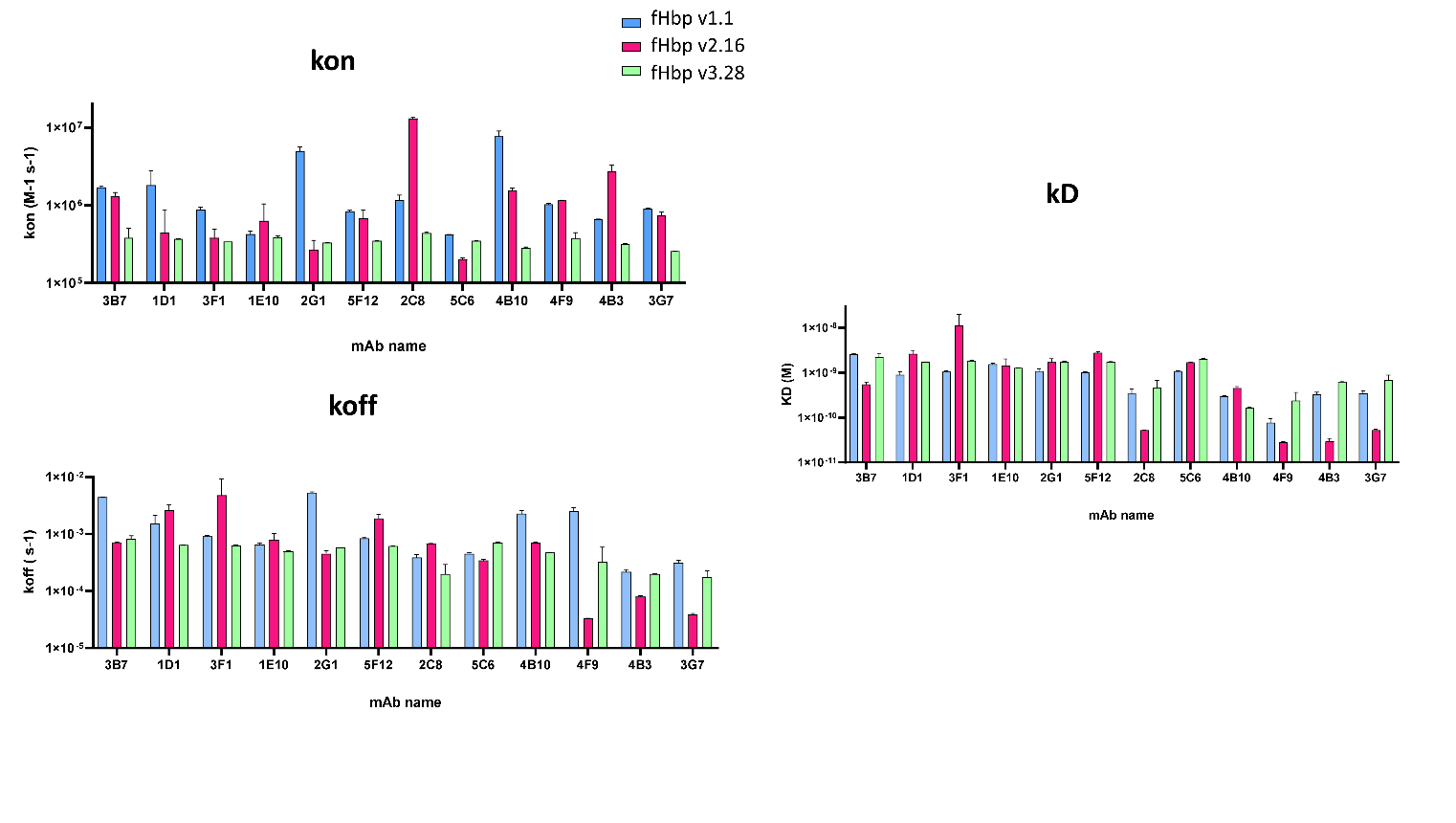
**

Supplement: S1 Fig — Data are shown for all twelve cross-reactive mAbs. (DOCX) [file ppat.1008882.s001.docx]

**S2 Figure**

**
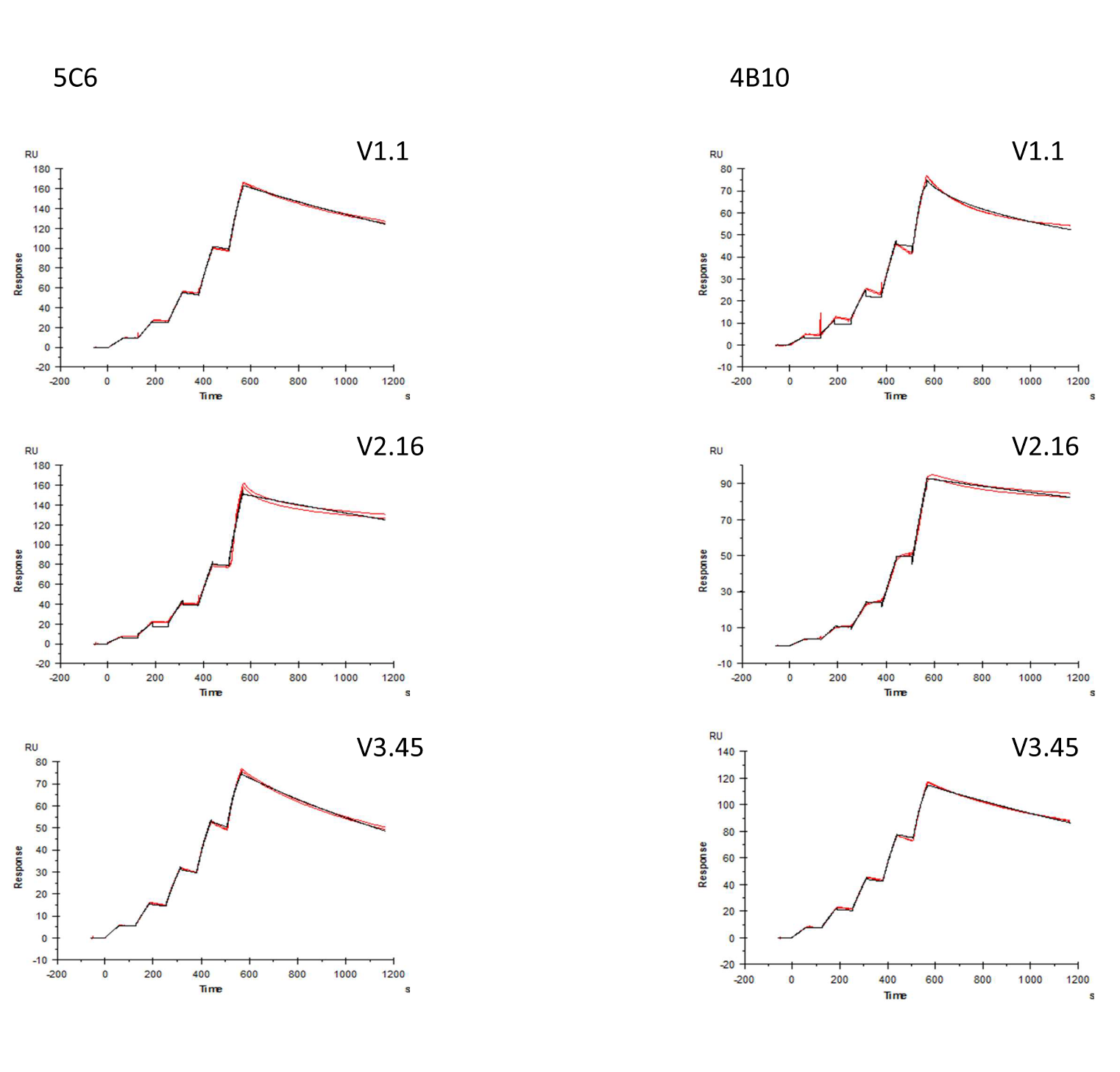
**

Supplement: S2 Fig — (DOCX) [file ppat.1008882.s002.docx]

**S3 Figure**

**
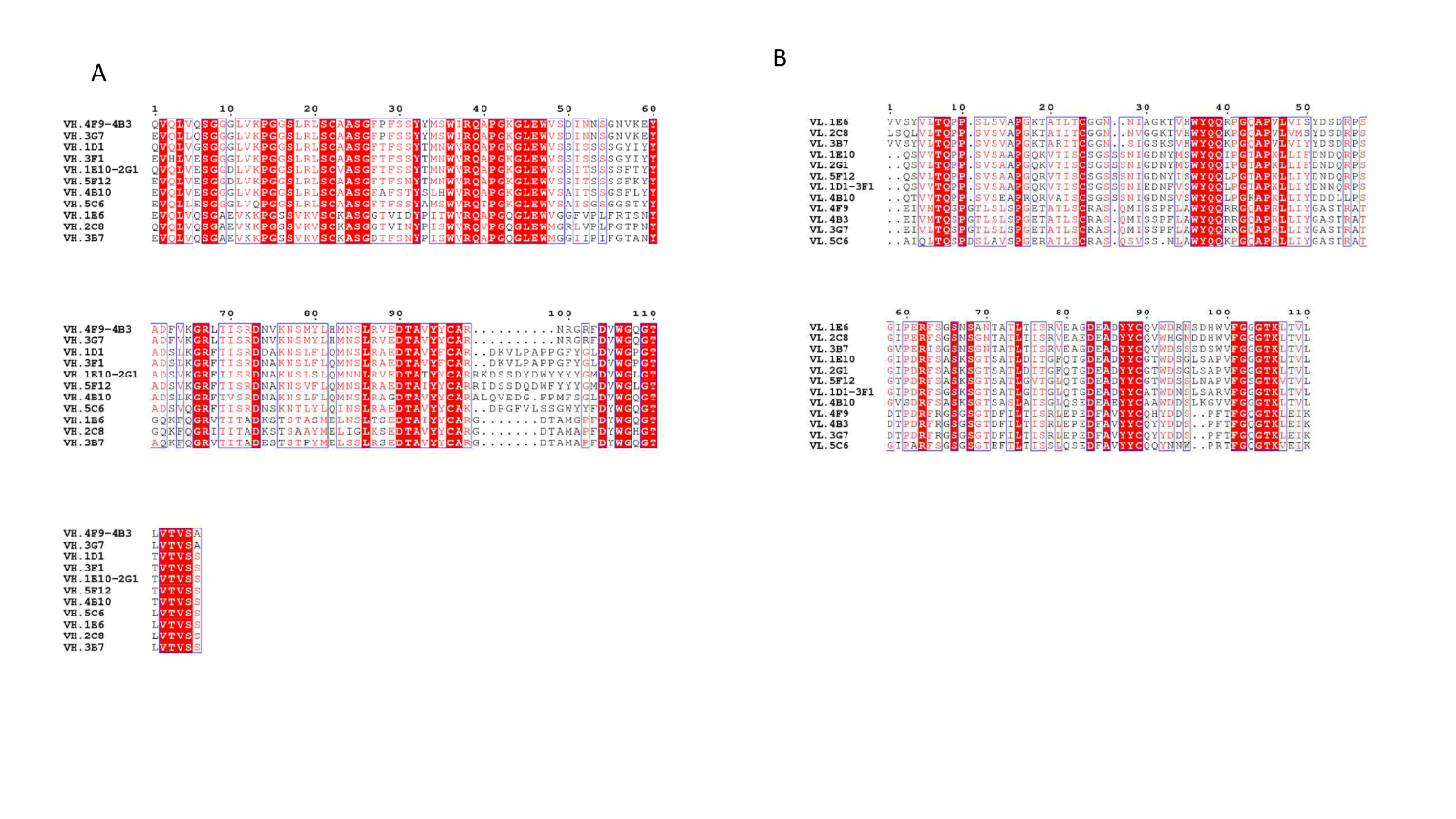
**

Supplement: S3 Fig — mAbs 4F9 and 4B3 and mAbs 1E10 and 2G1 have the same VH chain, while mAbs 1D1 and 3F1 have the same VL chain. (a) The nucleotide sequence alignment of VL chains, revealed that they differ by only for five substitutions overall, of which only the positions 9 and 271 introduce different residues within the mAbs. Remarkably, the substitution of thymine 271 in cytosine occurs in the CDR3 replacing the tyrosine on 4B3 and 3G7 with histidine on 4F9. (b) VH chains show five nucleotide substitutions. Only mutation in position 13 introduces a valine residue in 4B3 and 3G7 mAbs, while in 4F9 there is a leucine. The sequence alignment was performed with ClustalW and further represented using ESPript [46]. (DOCX) [file ppat.1008882.s003.docx]

**S4 Figure**


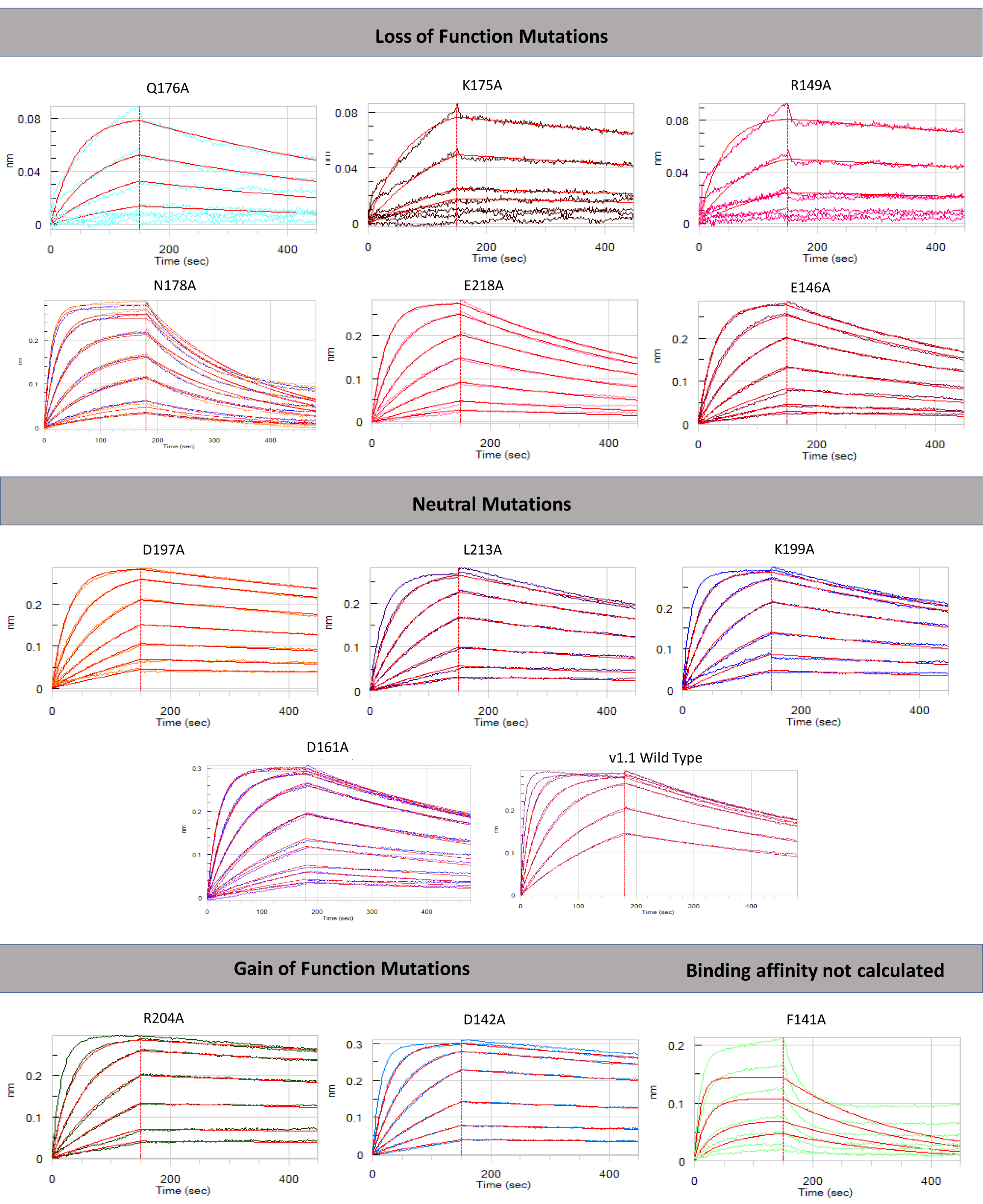

Supplement: S4 Fig — (DOCX) [file ppat.1008882.s004.docx]

**S4 Table.**

Degree of conservation of the key residues involved in fHbp v1 and mAb 4B3 binding


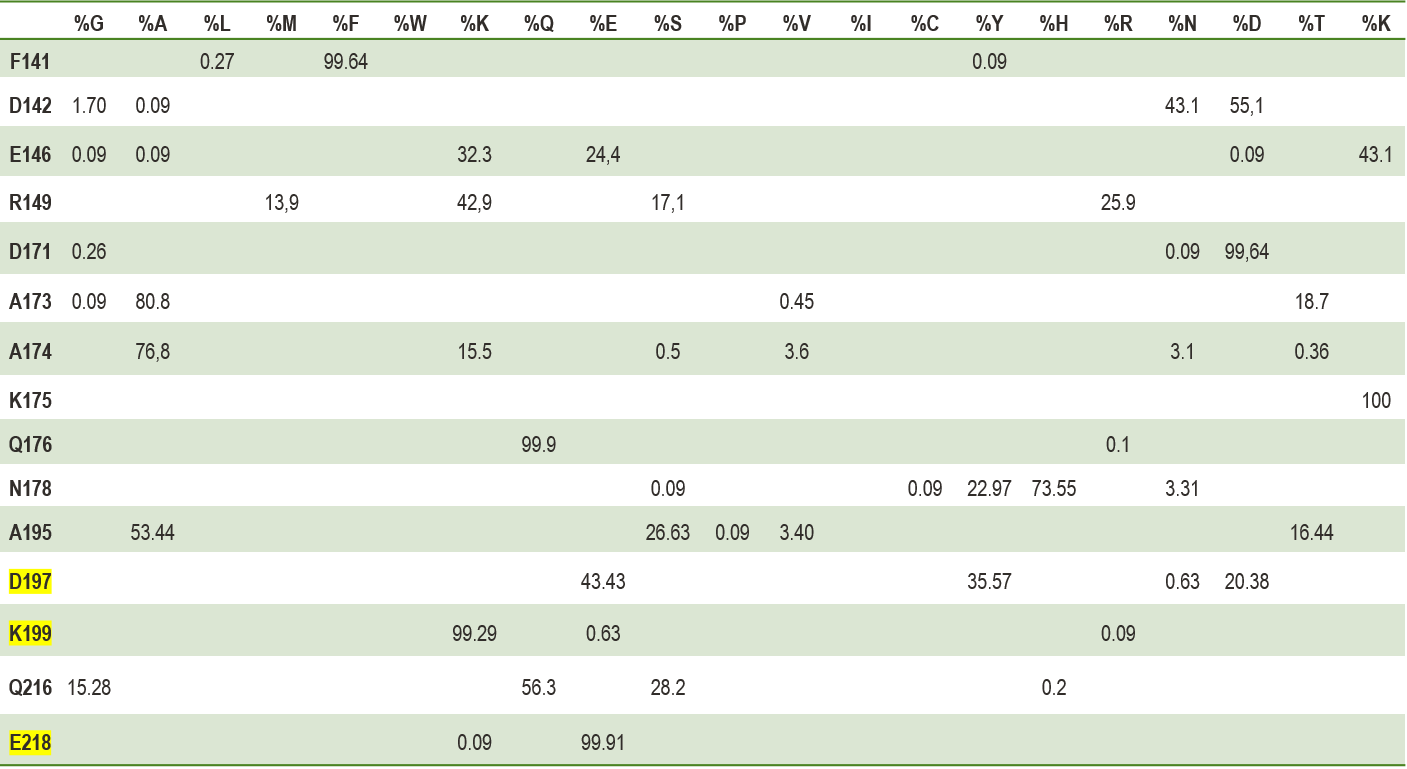

Supplement: S4 Table — The table shows the degree of conservation of the key residues of the mAb 4B3 epitope between fHbp sequences repertoire accessible in the Neisseria Meningitidis multilocus sequence typing (MLST) database at the website to https://pubmlst.org/neisseria which includes 1119 alleles of fHbp. (DOCX) [file ppat.1008882.s008.docx]
