# Supplementary material for: 4CMenB vaccine induces elite cross-protective human antibodies that compete with human factor H for binding to meningococcal fHbp
Source: PLoS Pathog. 2020 Oct 2;16(10):e1008882. doi: 10.1371/journal.ppat.1008882 (PMC7556464; doi:10.1371/journal.ppat.1008882)
Supplement: S1 Table — Data are shown for all twelve cross-reactive mAbs. (DOCX) [file ppat.1008882.s005.docx]

**S1 Table**

|  | **fHbp v1.1** | | |
| --- | --- | --- | --- |
| **mAbs** | **k*_on_* (M^-1^ s^-1^)** | **k*_off_* ( s^-1^)** | **KD (M)** |
| **3B7** | 1,71E+6 ± 0,02 | 4,42E-3 ± 0,03 | **2,6E-9 ± 0,06** |
| **1D1** | 1,81E+6 ± 0,67 | 1,54E-3 ± 0,04 | **8,93E-10 ± 1,03** |
| **3F1** | 8,85E+5 ± 0,49 | 9,17E-4 ± 0,15 | **1,04E-9 ± 0,05** |
| **1E10** | 4,27 E+5 ± 0,26 | 6,57E-4 ± 0,19 | **1,54 E-9 ± 0,05** |
| **2G1** | 4,51E+6 ± 0,48 | 5,0E-3 ± 0,01 | **1,08E-10 ± 0,08** |
| **5F12** | 8,54E+5 ±0,13 | 8,46E-4 ± 0,07 | **1,01E-9 ± 0,02** |
| **2C8** | 1,16E+6 ± 0,13 | 3,88E-4 ± 0,37 | **3,4E-10 ± 0,68** |
| **5C6** | 4,2E+5± 0,02 | 4,54E-4 ± 0,09 | **1,08E-9 ± 0,02** |
| **4B10** | 7,7E+6 ± 0,9 | 2,5E-3 ±0,2 | **2,96E-10 ± 0.08** |
| **4F9** | 3,29E+7 ± 0,19 | 2,51E-3 ± 0,03 | **7,7 E-11 ± 1,2** |
| **4B3** | 6,61E+5 ± 0,02 | 2,17E-4 ± 0,15 | **3,28E-10 ± 0,25** |
| **3G7** | 9,03E+5 ±0,03 | 3,11E-4 ± 0,25 | **3,44E-10 ± 0,32** |

|  | **fHbp v2.16** | | |
| --- | --- | --- | --- |
| **mAbs** | **k*_on_* (M^-1^ s^-1^)** | **k*_off_* ( s^-1^)** | **KD (M)** |
| **3B7** | 1,32E+6 ± 0,10 | 7,13E-6 ± 0,01 | **5,51E-10 ± 0,49** |
| **1D1** | 4,5E+5 ± 3,0 | 2,64E-3 ± 0,04 | **2,61E-9 ± 0,37** |
| **3F1** | 3,82E+5 ± 0,79 | 4,78E-3 ± 0,03 | **1,13E-8 ± 0,06** |
| **1E10** | 6,3E+5 ± 2,9 | 7,9E-4 ± 1,6 | **1,4E-9 ± 0,4** |
| **2G1** | 2,7E+5 ± 0,6 | 4,5E-4 ± 0,4 | **1,7E-9 ± 0,2** |
| **5F12** | 6,9E+5 ± 1,3 | 1,88E-3 ± 0,02 | **2,75E-9 ± 0,15** |
| **2C8** | 1,31E+7 ± 0,02 | 6,89E-4 ± 0,09 | **5,25E-11 ± 0,03** |
| **5C6** | 2,04E+5 ± 0,05 | 3,45E-4 ± 0,08 | **1,7E-9 ± 0,01** |
| **4B10** | 1,57E+7 ± 0,07 | 7,06E-4 ± 0,09 | **4,51E-11 ± 0,25** |
| **4F9** | 1,16E+6 ± 0,01 | 3,30E-5 ±0,01 | **2,85E-11 ±0,02** |
| **4B3** | 2,73E+6 ± 0,34 | 7,98E-5 ± 0,20 | **2,9E-11 ± 0,35** |
| **3G7** | 7,41E+5 ± 0,52 | 3,89E-5 ± 0,11 | **5,26 E-11 ± 0,22** |

|  | **fHbp v3.28** | | |
| --- | --- | --- | --- |
| **mAbs** | **k*_on_* (M^-1^ s^-1^)** | **k*_off_* ( s^-1^)** | **KD (M)** |
| **3B7** | 3,82 E+5 ± 0,85 | 8,3E-4 ±0,7 | **2,2E-9 ± 0,3** |
| **1D1** | 3,71E+5 ± 0,03 | 6,42E-4 ± 0,03 | **1,72E-9 ± 0,01** |
| **3F1** | 3,4 E+5 ± 0,8 | 6,3E-4 ± 3,2 | **1,80E-9 ± 0,01** |
| **1E10** | 3,9E+5 ± 0,1 | 4,95E-4 ± 0,09 | **1,27E-9 ± 0,02** |
| **2G1** | 3,32E+5 ± 0,01 | 5,84E-4 ± 0,01 | **1,75E-9 ± 0,01** |
| **5F12** | 3,50E+5 ± 0,01 | 6,13E-3 ± 0,02 | **1,75E-9 ± 0,01** |
| **2C8** | 4,3E+5 ± 0,1 | 2,0E-4 ± 0,6 | **4,6E-10 ± 1,6** |
| **5C6** | 3,50E+5 ± 0,01 | 7,10E-4 ± 0,07 | **2,03E-9 ± 0,02** |
| **4B10** | 2,84E+5 ± 0,06 | 4,73E-4 ± 0,01 | **1,67E-9 ± 0,03** |
| **4F9** | 3,8 E+5 ± 0,5 | 9,5E-4 ± 4,3 | **2,4E-10 ± 0,9** |
| **4B3** | 3,15E+5 ± 0,06 | 2,00E-4 ±0,01 | **6,33E-10 ± 0,13** |
| **3G7** | 2,58E+5 ± 0.01 | 1,76E-4 ± 0,35 | **6,8E-10 ± 1,4** |
