# Supplementary material for: 4CMenB vaccine induces elite cross-protective human antibodies that compete with human factor H for binding to meningococcal fHbp
Source: PLoS Pathog. 2020 Oct 2;16(10):e1008882. doi: 10.1371/journal.ppat.1008882 (PMC7556464; doi:10.1371/journal.ppat.1008882)
Supplement: S2 Table — The results in bold, in the first column of each strain, were used for the histograms in Fig 2. (DOCX) [file ppat.1008882.s006.docx]

**S2 Table**

|  | **rSBA titers** | | | | | | **hSBA titers** | | | | | |
| --- | --- | --- | --- | --- | --- | --- | --- | --- | --- | --- | --- | --- |
|  | **fHbp v1** | | **fHbp v2** | | **fHbp v3** | | **fHbp v1** | | **fHbp v2** | | **fHbp v3** | |
| **5F12** | **<16** | <4 | **128** | 64 | **<16** | <16 | **<4** | <4 | **<4** | <4 | **<4** | <4 |
| **5C6** | **512** | 512 | **256** | 128 | **<16** | <16 | **<4** | <4 | **<4** | <4 | **<4** | <4 |
| **4B10** | **<16** | <16 | **2048** | 512 | **<16** | <16 | **<4** | <4 | **<4** | <4 | **<4** | <4 |
| **4F9** | **8192** | 8192 | **8192** | >8192 | **8192** | >8192 | **128** | 64 | **512** | >=512 | **2048** | 512 |
| **4B3** | **8192** | 16384 | **16384** | >8192 | **8192** | >=8192 | **128** | 1024 | **1024** | >=512 | **2048** | 1024 |
| **3G7** | **4096** | 8192 | **8192** | >=8192 | **8192** | >=8192 | **64** | 64 | **1024** | >=512 | **1024** | 256 |
