# Supplementary material for: 4CMenB vaccine induces elite cross-protective human antibodies that compete with human factor H for binding to meningococcal fHbp
Source: PLoS Pathog. 2020 Oct 2;16(10):e1008882. doi: 10.1371/journal.ppat.1008882 (PMC7556464; doi:10.1371/journal.ppat.1008882)
Supplement: S3 Table — Each Tm value provided is the average of two independent experiments. (DOCX) [file ppat.1008882.s007.docx]

**S3 Table**

|  | fHbp Mutation | T_m_ (°C) |
| --- | --- | --- |
| Loss of Function Mutations | Q176A | 84.9 |
|  | K175A | 78.6 |
|  | R149A | 84.3 |
|  | N178A | 87.5 |
|  | E218A | 85.6 |
|  | E146A | 87.7 |
| Neutral Mutations | D161A | 84.8 |
|  | L213A | 82.8 |
|  | D197A | 87.8 |
|  | K199A | 83.7 |
|  | Wild Type | 86.9 |
| Gain of Function Mutations | D142A | 79.9 |
|  | R204A | 81.4 |
| K_D_ Not Determined | F141A | 75.0 |
